# Taxonomy of Chemical Bondings: Opportunities and Challenges

**DOI:** 10.1002/anie.202506525

**Published:** 2025-05-22

**Authors:** Andrea Pizzi, Giancarlo Terraneo, Cristina Lo Iacono, Roberta Beccaria, Arun Dhaka, Giuseppe Resnati

**Affiliations:** ^1^ NFMLab, Department of Chemistry, Materials, Chemical Engineering “Giulio Natta” Politecnico di Milano Via Mancinelli 7 Milano 20131 Italy

**Keywords:** Chemical bonds, Chemical interactions, Nomenclature, Taxonomy, Terminology

## Abstract

The concept of the chemical bond is fundamental to chemistry, governing atomic interactions that define all known matter. Despite this central role, the classification and most convenient naming of chemical bonds remain subjects of debate due to the diverse theoretical models and experimental observations. Modelings from quantum mechanical calculations and heuristic principles from experimental observations offer valuable and complementary insights, but sometimes the match and coalescence of these different approaches into a common terminology is not immediate. This paper describes a hierarchical categorization of noncovalent interactions based on the electrophilic atom involved, aligning with IUPAC definitions of hydrogen bonding (HB), halogen bonding (HaB), chalcogen bonding (ChB), and pnictogen bonding (PnB). The resulting taxonomy may avoid some ambiguities that arise from naming interactions based on single chemical/physical features. The proposed categorization that moves from more general and comprehensive terms to more specific and descriptive terms may ensure clarity, comprehensiveness, consistency with periodic trends, and invariancy over evolving understanding of the chemical bonds so that findings can be communicated and stored effectively via both human and machine based protocols.

## Graphical abstract

The recent focus on supramolecular and nanosized systems produced a major increase in the types of observed noncovalent interactions and in the diversity of terms used to designate them. A hierarchical categorization (taxonomy) of terms used to designate electrophile···nucleophile interactions is proposed here. Core elements of this classification are some recent IUPAC definitions referring to the electrophile.

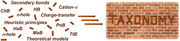

## 1 Introduction

*If names be not correct*,

*language is not in accordance with the truth of things* ^[^ ^1^ ^]^

Confucius’ statement paraphrased in the Chinese proverb:

*The beginning of wisdom is to call things by their proper name*

The chemical bond has been^[^ ^2^, ^3^, ^4^, ^5^, ^6^ ^]^ and is^[^ ^7^, ^8^, ^9^, ^10^, ^11^ ^]^ at the centre stage in chemistry. This is no surprise as any chemical process involves the formation and cleavage of either strong or weak bonds between atoms.^[^ ^12^ ^]^ Virtually, all the organic and inorganic matter we experience daily is composed by bonded atoms, not by isolated ones. Although the matter in the universe is compounded by a relatively small number of chemical elements, the variety of the polyatomic aggregates is enormous. It spans from the diatomic gases, to the large biomacromolecules and the metallic entities. Such a diversity is associated with, and enabled by, an enormous diversity of chemical bondings.

This variety of different chemical bonds is studied by using many different methodologies and this favors the multiplying of models for describing bonds and of terms for naming them. In the past century and in the present one, the research on the chemical bond has been characterized by a dual pathway, experimental studies, and theoretical analyzes. These approaches typically afford pictures of bonds whose correspondence and match may not be immediate. G. Frenking observed that different definitions of bonds exist and they exemplify the differences between the approaches used by chemistry and physics to describe the world.^[^ ^13^ ^]^ The nonstraightforward match between the bond representations, based on physically observable quantities and on quantum chemical or orbital calculations,^[^ ^14^, ^15^, ^16^ ^]^ generated discussions, if the most convenient names of the bonds have to relate preferentially to one approach or the other.^[^ ^17^, ^18^, ^19^, ^20^, ^21^, ^22^, ^23^ ^]^

At the same time, the strive for connecting the two pathways has been highly rewarding as succeeded in bridging the heuristic principles developed from the regularities in an enormous number of experimental observations and the equally enormous numerical output of computational analyzes. The results of this interfacing are a variety of symbols, models, classifications, and terminologies that are rich in sophisticated information, very useful, and commonly used, though sometimes they are poorly defined. Typically, the two pathways have been interfaced via somewhat arbitrary interpretative receipts and informed guesses rather than rigorously deductive and formal thinking. G. Frenking's statement that “Chemistry as it is currently applied and understood can be considered as the science of fuzzy concepts”^[^ ^9^, ^24^, ^25^ ^]^ describes iconically the true spirit of chemistry. A fuzzy character also directly impacts on the present understanding of the chemical bond, on the used chemical terminology, and on the controversies if the most convenient terms have to relate preferentially to experimental or theoretical models. In his essay on the IUPAC definition of the hydrogen bond, G. R. Desiraju wrote “A possible reason for all this disputation about nomenclature is that chemistry still remains a gloriously qualitative subject”.^[^ ^26^ ^]^ S. Shahbazian keenly noted that “Quantum mechanics is the best example demonstrating the fact that generally, formalism does not in itself impose an interpretation. In other words, formalism may be compatible/coexist with a number of interpretations”.^[^ ^27^ ^]^ Moreover, models of bonds between atoms have been sometimes mistaken for the “real bond” in the matter and this opened the way to a further increase in the variety of interpretations, terminologies, and controversies to describe them.

Given this diversity of the bonds present in the matter and of the languages used to describe them, it can be easily understood why surprising or even paradoxical or contradictory statements have been given in authoritative papers on the chemical bond. As to surprising statements, G. Desiraju wrote that “the word bond has an almost religious connotation for chemists. It is this word that confers on us an identity that distinguishes us from all other scientists. Every chemist has his or her own idea as to what constitutes a bond”.^[^ ^26^ ^]^ Despite the momentous results of theoretical chemistry, S. Shahbazian wrote “there is no general theoretical scheme that may claim to encompass the description of all known types of chemical bonds.”^[^ ^27^ ^]^ As to paradoxical statements,^[^ ^28^ ^]^ H. Jacobsen spoke about “the dilemma of a proper description of a bond between atoms” and arrived to concluding that “it has become clear that the chemical bond per se does not exist. Each bond possesses its very own specific character”.^[^ ^29^ ^]^ Differently, J. C. Slater wrote “there is no very fundamental distinction between the van der Waals binding and covalent binding”^[^ ^30^ ^]^ and R. H. Crabtree stated “there is a close relationship between hydrogen bonding (HB), hypervalent bonding (HVB), and secondary bonding (SB): although often considered in isolation, they have key bonding aspects in common”.^[^ ^31^ ^]^ Shifting to contradictory statements, it may be reminded that a bond critical point (BCP) is typically found in a chemical bond and D. Stalke wrote that a BCP “is a necessary and sufficient condition for the chemical bond”.^[^ ^28^ ^]^ But J. S. Murray wrote that ″bond critical points are neither necessary nor sufficient for attractive interactions, … and in some instances also pointing to repulsive interactions″.^[^ ^32^ ^]^ Statements similar to Murray's have been made by others.^[^ ^33^, ^34^ ^]^ Interestingly, R. F. W. Bader published in 1998 a paper entitled “A Bond Path: A Universal Indicator of Bonded Interactions”^[^ ^35^ ^]^ and in 2009 another paper entitled “Bond Paths Are Not Chemical Bonds”.^[^ ^36^ ^]^ This was his own way to say that a bond between atoms is not necessarily a chemical bond.^[^ ^37^ ^]^ Indeed, statements about the chemical bond may be seemingly or implicitly conflicting with each other as far as both conceptual and semantic aspects is concerned. Notwithstanding the diversity of statements exemplified above, it seems there is a consensus on the existence of several different types of chemical bonds.

**Figure 1:**
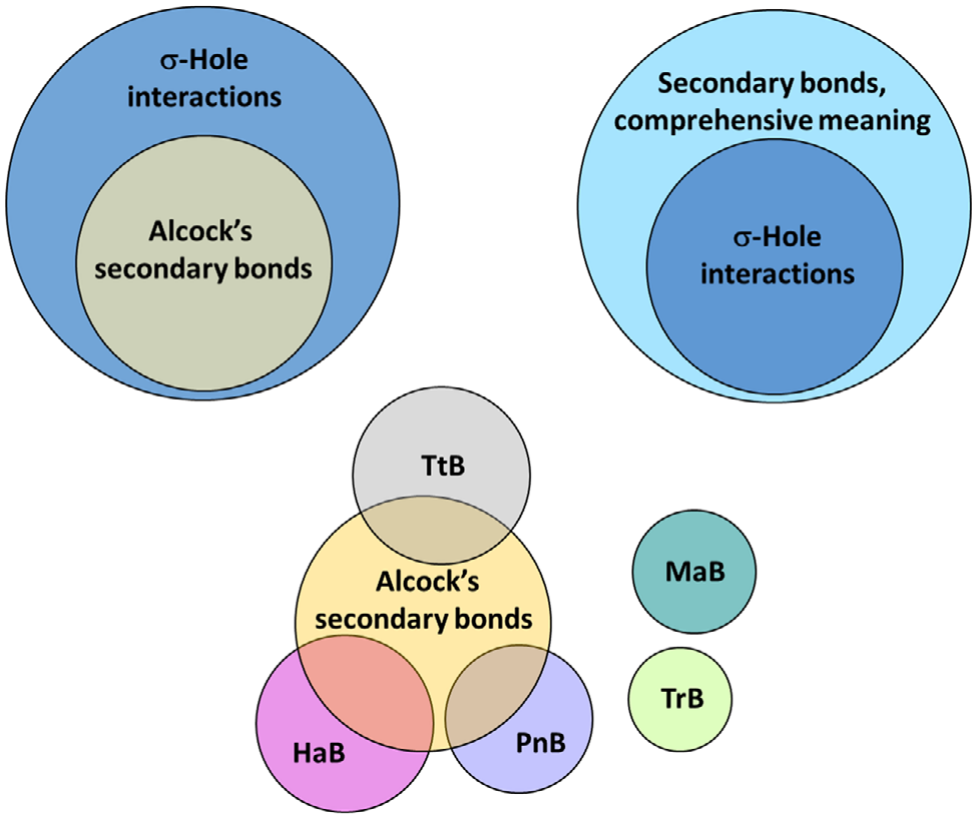
Venn notation of the relationship between different sets of interactions. Top: the secondary bonds according to Alcock's meaning^[^ ^54^ ^]^ (Section 3.1) are a subset of the σ‐hole interactions,^[^ ^43^, ^44^, ^45^, ^46^, ^47^, ^48^ ^]^ which, in their turn, are a subset of the secondary bonds when the term is used according to the comprehensive meaning.^[^ ^55^ ^]^ Bottom: the tetrel bond (TtB),^[^ ^56^, ^57^ ^]^ the pnictogen bond (PnB),^[^ ^42^ ^]^ and the halogen bond (HaB)^[^ ^40^ ^]^ (see Sections 2.3 and 2.4) are sets of interactions intersecting with the set of the secondary bonds according to Alcock's meaning, whereas the matere bond (MaB)^[^ ^58^ ^]^ and the triel bond (TrB)^[^ ^59^, ^60^ ^]^ (Sections 2.5) are disjoint sets.

According to Wittgesnstein's stance in the Tractatus Logico‐Philisophicus,^[^ ^38^ ^]^ in any communication (and we add especially in scientific communication), a preference should be given to words that have a logical relationship with objects they stand for and it is fundamental to provide an account of the logic underlying this relationship. Consequently, a preference should be given to a terminology wherein bonds and sets of bonds are profiled by their names. If so, the key features of a given bond can be easily surmised from its name and it is immediately apparent if one set of bonds is a subset of another set or if two sets are intersecting or disjoint^[^ ^39^, ^40^, ^41^, ^42^, ^43^, ^44^, ^45^, ^46^, ^47^, ^48^, ^49^, ^50^, ^51^, ^52^, ^53^ ^]^ (Figure 1). In other words, it is better if the name designating a bond explicitly relates to the most distinctive feature(s) characterizing that bond. In this way, the name immediately communicates these feature(s) and ambiguity or confusion with other bonds is avoided. Similarly, it is important that the name used to designate a set of bonds is unambiguously associated with the key commonalities shared by the bonds of the set, whatever these commonalities are. If so, the boundaries of each set are unequivocally defined and ambiguity or confusion are avoided once again.

Due to the enormous diversity of the chemical bonds that mirrors in the enormous diversity of the terms used to name them, it becomes a terrific challenge to identify the features that are most distinctive of a single bond and the same holds for the key commonalities shared by the bonds of a set of bonds. To reach a consensus on these aspects is an equally terrific challenge, but it is instrumental for a good communication. A “common understanding of commonly used language”^[^ ^29^ ^]^ is crucial for the advancement of science as it allows for information to be efficiently accumulated and knowledge to be effectively managed.

**Figure 2:**
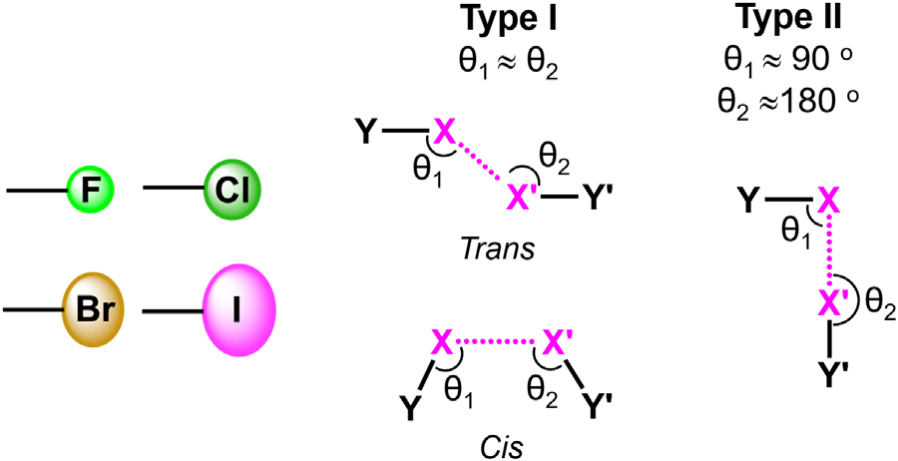
Geometric features of Type I and Type II halogen···halogen interactions.

The attention to supramolecular and nanosized systems grew enormously in the last decades, and this produced a parallel growth of the interest in the weak bonds. H. J. Schneider started his review on the binding modes in supramolecular complexes stating that “With courageous simplification, one might assert that the chemistry of the last century was largely the chemistry of covalent bonding, whereas that of the present century is more likely to be the chemistry of noncovalent binding”.^[^ ^11^ ^]^ The upsurge of studies in the field produced a further increase in the diversity of observed and characterized weak bonds. New terms and phrasings have been introduced in order to describe the new findings and to manage the increased complexity in the field. The correlated, electrostatic, dispersion, mixed, polar, and charge‐shift bonds are itemized in a paper entitled “Distinguishing bonds”, wherein M. Rahm proposes criteria for describing bonds character^[^ ^61^ ^]^ and many other bond names and classifications could be listed. For instance, in 1986, R. Parthasarathy designated the short contacts between halogen atoms as halogen···halogen interactions.^[^ ^62^ ^]^ In 1989, these contacts were classified as Type I or Type II halogen···halogen interactions as a function of their geometries.^[^ ^63^ ^]^ Specifically, the two angles, *θ*_1_ and *θ*_2_, around the halogen atoms in the adduct Y─X···X’─Y’(Y/Y’═C, F, Cl, Br, I, …; X/X’═F, Cl, Br, I; *θ*_1_ = ∠Y─X···X’; *θ*_2_ = ∠Y’─X’···X) are similar in Type I, whereas θ_1_ ≈ 90° and θ_2_ ≈ 180° in Type and θ_2 _≈ 90° in Type II (Figure 2). This classification has been, and continues to be, extensively used. Twenty years later, the Type I interactions were split into *cis* and *trans* sub‐sets,^[^ ^64^, ^65^ ^]^ and after additional ten years, Type III and IV interactions were introduced, once again moving from geometric features.^[^ ^66^, ^67^ ^]^ As discussed above, the kaleidoscopic diversity of the terms used to designate the chemical bonds and their sets is justified by the diversity of the bonds present in the matter and by the variety of the methodologies used for their study. It is important to remind that terms also differ from each other in the logical relationship to the corresponding bonds; this relationship being more robust for some terms and less robust for some others. For some terms, the relationship strictly depends on the model used for bond understanding, for some other terms the relationship has changed over time, in connection with the change in the understanding of bonds (see Sections 2 and 3). Moreover, some terms are highly suited to be organized hierarchically with other terms as a function of their respective degrees of generality and this offers an additional opportunity for an effective, general, and unambiguous communication. For some other terms, a hierarchical organization and an identification of the relationship between the respective sets is more problematic.

In this paper, we will discuss a hierarchical classification of some terms used to indicate weak chemical bonds (namely interactions).^[^ ^12^ ^]^ These terms were selected among those commonly used in the literature because: i) their logical relationship to the bonds they stand for is particularly robust and invariant with respect to the evolving understanding of interactions, and ii) they are particularly suitable for constructing a hierarchical organization of chemical interactions.

Many interactions are formed thanks to the attractions between an atom, or group of atoms, which act as electrophile/Lewis acid and an atom, or group of atoms, which act as nucleophile/Lewis base. This polar behaviour can operate at different levels of generality and is used here to classify many distinct weak bonds that are grouped into sets and subsets identified by their different degree of comprehensiveness, namely, as a function of shared characteristics having different levels of comprehensiveness.^[^ ^68^, ^69^, ^70^ ^]^ Specifically, moving from the IUPAC defined halogen bond (HaB),^[^ ^40^ ^]^ chalcogen bond (ChB),^[^ ^41^ ^]^ pnictogen bond (PnB),^[^ ^42^ ^]^ the naming of interactions by referring to the group of the electrophilic atom is proposed as the core of a systematic categorization (taxonomy) of a variety of weak bonds.

For instance, the HaB, ChB, PnB, and analogous interactions, e.g., the tetrel bond (TtB),^[^ ^56^, ^57^ ^]^ will be discussed with respect to more comprehensive sets of bonds, e.g., the closed‐shell,^[^ ^71^, ^72^, ^73^ ^]^ secondary,^[^ ^55^, ^74^, ^75^, ^76^ ^]^ and charge‐tranfer^[^ ^77^, ^78^ ^]^ interactions, as well as to the σ‐^[^ ^43^, ^44^, ^45^, ^46^, ^47^, ^48^ ^]^ and π‐hole bonds.^[^ ^49^, ^50^, ^51^ ^]^ The HaB, ChB, PnB, and TtB will be discussed also with respect to less comprehensive sets, e.g., it will be considered how the chlorine bond,^[^ ^79^, ^80^, ^81^ ^]^ selenium bond,^[^ ^82^ ^]^ and carbon bond^[^ ^83^, ^84^, ^85^ ^]^ are subsets of the HaB, ChB, and TtB.

This consistent and hierarchical categorization will allow for filing a taxonomy of chemical interactions. The success of the taxonomic classification in organizing the enormous diversity of living organisms prompted the use of taxonomy in many other fields of science.^[^ ^86^, ^87^, ^88^, ^89^, ^90^, ^91^, ^92^ ^]^ A taxonomy of chemical interactions may allow for organizing in a consistent and hierarchical way the enormous diversity of weak chemical bonds. Benefits can arise in any field where chemical bondings are addressed. This taxonomy may also be a step ahead toward a “common understanding of commonly used language”^[^ ^29^ ^]^ so that the chemical bondings and their differences and similarities can be debated straightforwardly and unequivocally.

## 2 Naming Interactions From the Electrophilic Site

*The first requisite of an ideal language would be that there*

*should be one name for every simple, and never the same name*

*for two different simples*

B. Russel^[^ ^93^ ^]^

*One name stands for one thing, and another for another thing*,

*and they are connected together. And so the whole, like a living*

*picture, presents the atomic fact (4.0311)*

L. Wittgenstein^[^ ^94^ ^]^

The multiplicity of terminologies in the field of the weak bonds may lead to think that no way out can be found to the problems resulting from the “incommensurable jargon of different paradigms”.^[^ ^95^ ^]^ But fortunately phenomena presented with different wordings often have common features.

An Ariadne's thread can be found on the fact that many interactions consist in the attraction between electrophilic atoms or groups of atoms (namely, sites with depleted electron density, and in most cases, with a positive surface electrostatic potential) and nucleophilic atoms or groups of atoms (namely, sites with excess electron density, and in most cases, with a negative electrostatic potential). The hydrogen bond (HB)^[^ ^52^, ^53^ ^]^ is probably the most studied and impacting interaction and as such it is a reference when a classification of chemical interactions is pursued. As reiterated in the IUPAC definition of the HB issued in 2011,^[^ ^52^, ^53^ ^]^ the hydrogen atom is the electrophile. To name interactions by referring to the electrophile is thus a well‐established practice. This approach has been adopted by the IUPAC recommendations of the HaB,^[^ ^40^ ^]^ ChB,^[^ ^41^ ^]^ and PnB,^[^ ^42^ ^]^ which defined these interactions as the bonds that electrophilic atoms, belonging to groups 17, 16 and 15 of the periodic table, form with nucleophiles. In the last 40 years, these bonds received a particular attention (see Sections 2.3 and 2.4) and IUPAC recommendations recognized their impact. These IUPAC defined terms are key parts of the categorization proposed here. Other key parts are the electrophile···nucleophile interactions wherein the electrophilic atom belongs to the remaining groups of the p block (e.g., tetrel bond (TtB)^[^ ^56^, ^57^ ^]^ and noble gas bond (NgB)).^[^ ^96^ ^]^ The same holds for some interactions, wherein d block elements are the electrophile (e.g., matere bond (MaB)^[^ ^58^ ^]^ and osme bond (OmB)).^[^ ^97^ ^]^

### 2.1 Two‐Atoms and One‐Atom Nomenclature

The atoms forming an interaction are very distinctive features of the interaction. They do not vary as a function of the model used for the interaction description or of any other factor (e.g., phase, conditions or environment wherein the interaction is formed, technique used for its study, etc.). A terminology referring to the atom(s) forming the interaction thus seems very convenient as the logical relationship between names and interactions is invariant.

Most bondings involve two atoms, some of them more than two (e.g., π–π and cation–π bonds). A commonly used notation of covalent and coordination bonds gives the symbols (or the names) of the bonded atoms spaced by a dash, e.g., C─N or carbon–nitrogen indicate a covalent bond between carbon and nitrogen. An analogous notation for many weaker bonds frequently substitutes three dots for the dash, e.g., H···N or hydrogen···nitrogen indicate a hydrogen bond wherein hydrogen is the acceptor of electron density.

This two atoms notation has a well‐defined relationship with the bond under consideration, but it has no relation to the nature or geometry of the designated bonds, i.e., the same notation encompasses interactions wherein nature or geometry are different. The halogen–halogen interactions^[^ ^62^ ^]^ mentioned above are a good example. These bonds can have quite different geometries; indeed, they have been divided into two^[^ ^63^ ^]^ or more^[^ ^64^, ^65^, ^66^, ^67^ ^]^ groups as a function of the angles *θ*_1_ and *θ*_2_ (Figure 2). Importantly, these different geometries are a consequence of the different forces driving the interactions occurrence.^[^ ^98^ ^]^ There is a consensus that the attractive nature of the Type II halogen–halogen interactions arises from the positive region (σ‐hole) of one halogen approaching the negative region (typically a lone pair) of the other interaction site. It is now commonly accepted that Type II halogen–halogen interactions are halogen bonds.^[^ ^99^ ^]^ The nature of Type I halogen–halogen interactions is much more disputed.^[^ ^100^ ^]^ It has been proposed Type I bonds do not arise from noncovalent bonding, rather they are geometry‐based contacts determined by close packing requirements.^[^ ^101^ ^]^ Alternatively, it has been speculated that a key role is played by the elliptic shape of halogen atoms^[^ ^102^ ^]^ or by dispersive forces.^[^ ^103^, ^104^ ^]^

**Figure 3:**
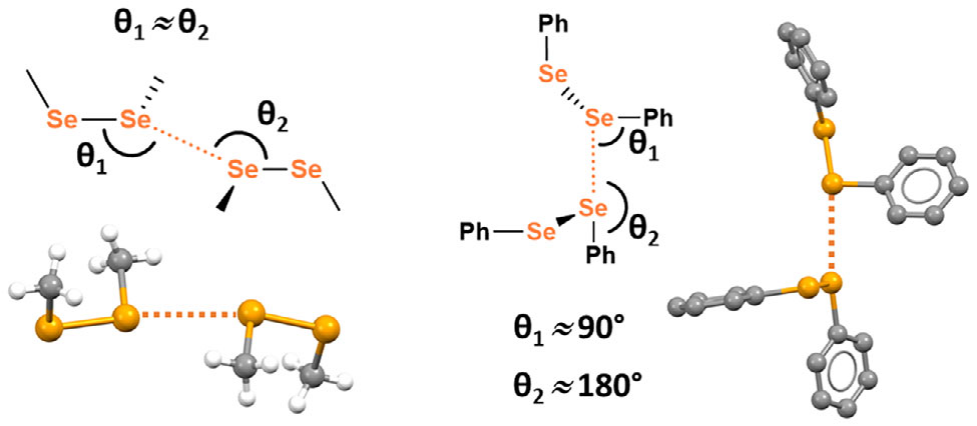
Geometric features of Type I and Type II chalcogen–chalcogen interactions in dimethyldiselenide and diphenyldiselenide.^[^ ^106^ ^]^

Other interaction names lack a one‐to‐one relation with the interaction geometry and nature. The term chalcogen–chalcogen interactions has been extensively used^[^ ^105^, ^106^, ^107^, ^108^ ^]^ to designate contacts wherein the diversity of the angles between the interaction and the covalent bonds formed by the chalcogen atoms (Figure 3) recalls that in the Type I and Type II halogen–halogen interactions.^[^ ^109^ ^]^ For instance, the term chalcogen–chalcogen interaction has been used for both the short Se⋯Se contact in dimethyldiselenide, which recalls the geometry of Type I halogen–halogen interactions, and for the short Se⋯Se contact in diphenyldiselenide, which recalls the geometry of Type II halogen–halogen interactions.

Analogously, the term hydrogen–hydrogen bonding has been used for linear interactions between identical hydrogen atoms that are close to electrical neutrality^[^ ^110^ ^]^ and for orthogonal interactions between hydridic and protonic hydrogens.^[^ ^111^ ^]^

**Figure 4:**
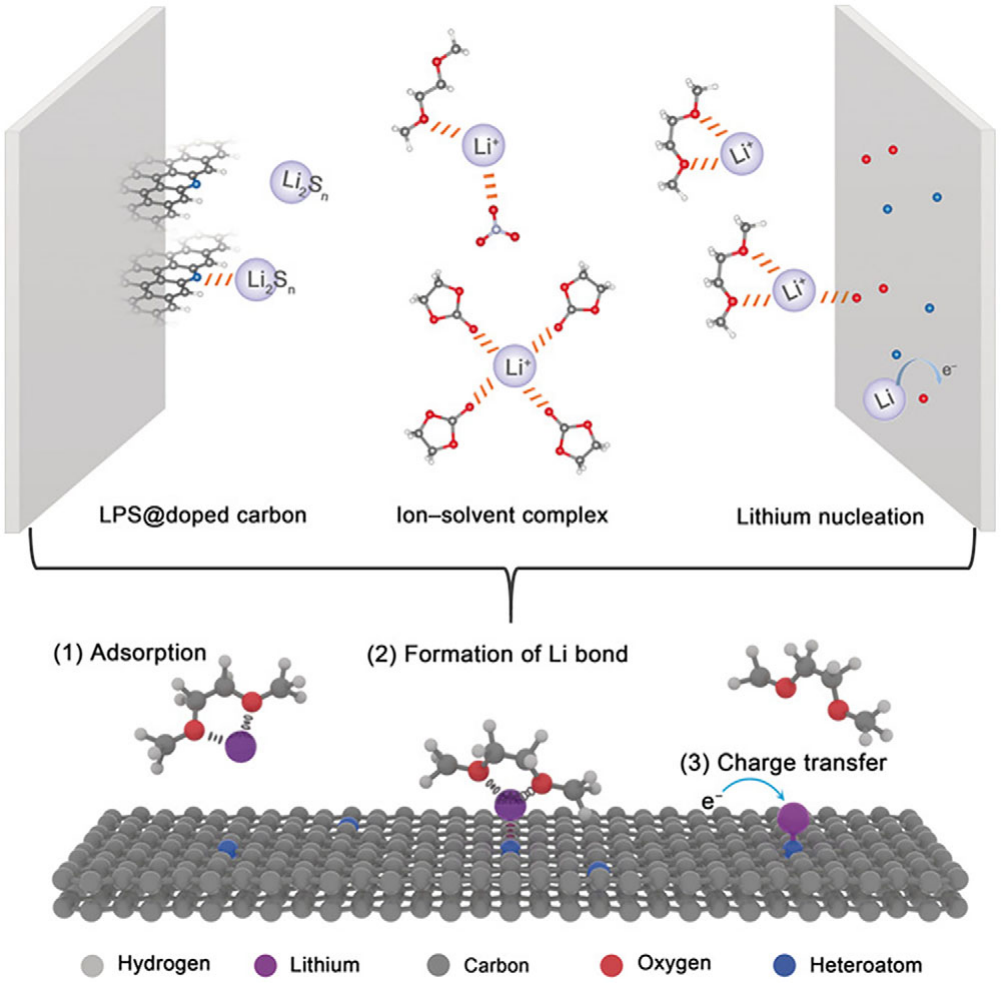
Top: schematic representation of the Li bond between lithium polysulfide (LPS) and N of the N‐doped graphene in lithium batteries. Bottom: representation of lithium nucleation on conductive frameworks (with copyright permission from John Wiley and Sons).

**Figure 5:**
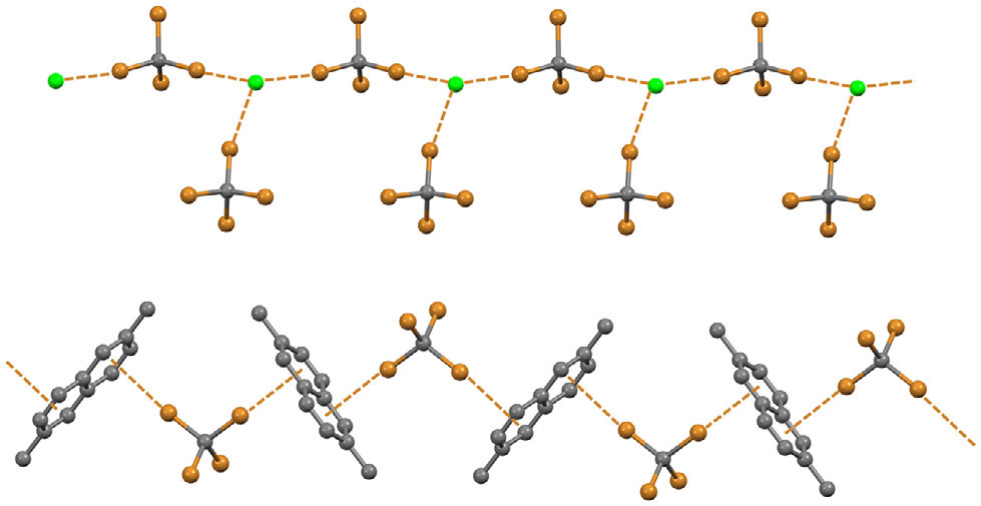
Partial representation of the infinite chains assembled by σ‐hole HaBs in *n*‐Pr_4_N^+^Cl^−^/CBr_4_ cocrystal (top, *n*‐Pr_4_N^+^ cations omitted) and by π‐hole HaBs in 2,6‐dimethylnaphtalene/CBr_4_ cocrystals^[^ ^117^ ^]^ (bottom, hydrogens omitted). HaBs are dashed brownish lines. Color code: carbon, grey; bromine, brownish; chlorine, green.

Several other commonly used names of weak bonds refer to only one of the involved atoms and they refer to the electrophilic atom consistent with the name of the hydrogen bond. Some early cases of this tendency dates back to 1970s when L. Allen published a paper entitled “The lithium Bond”, wherein calculations predicted that lithium in LiF can form attractive interactions with electron‐rich partners.^[^ ^112^ ^]^ Few years later, G. C. Pimentel confirmed experimentally that lithium bonds occur between LiCl or LiBr and various bases.^[^ ^113^ ^]^ Recently, it has been recognized that this bond has a role in lithium batteries (Figure 4).^[^ ^114^, ^115^ ^]^ Successively, many other bondings have been named referring to the electrophilic atom.^[^ ^79^, ^80^, ^81^, ^82^, ^83^, ^84^, ^85^ ^]^ For instance, Na^+^···π interactions have been named sodium bonds,^[^ ^116^ ^]^ the short contacts formed in the gas phase by Cl_2_ or ClF with π‐bond electrons and by CHBr_3_ or CBr_4_ with σ lone pairs or π‐bond electrons (Figure 5) were named chlorine bond^[^ ^79^, ^80^, ^81^ ^]^ and bromine bonds,^[^ ^117^ ^]^ respectively.

### 2.2 From Atom‐ to Group‐Nomenclature

The collective names referring to the group to which the electrophilic atom of an interaction belongs gained much greater acceptance than the interactions names referring to the single electrophilic atom. This possibly happened as the group nomenclature offers the advantage that a less numerous set of interactions names gets in use. Moreover, it allows for identifying the distinctive features of the interactions formed by an element via a comparison with the features of interactions involving other elements of the same group.

A very important prerequisite for the correct use of a nomenclature referring to the electrophile is the correct identification of the electrophilic atom in the interaction as this nomenclature implies and communicates a given geometry around that atom. The geometry around the electrophile is different from the geometry around the nucleophile, both being determined by the regions of electron density localization in an acceptor versus donor. If the nucleophilic atom is exchanged for the electrophilic one, the name of the interaction changes, and most importantly, this misnaming communicates wrong geometric features. Specifically, the geometry around the electrophile is attributed to the nucleophile and vice versa.

This has been the case both for interactions wherein nucleophilic halogens were interacting with electrophilic hydrogens^[^ ^118^ ^]^ and for interactions wherein electrophilic halogens were interacting with nucleophilic hydrogens.^[^ ^119^, ^120^, ^121^ ^]^ Specifically, hydrogen atoms bonded to more electronegative atoms (e.g., Z─H, Z═O, N, etc.) have a partial positive charge and tend to act as electrophiles. When these hydrogens interact with a monovalent chlorine (Z─H···X─T, X═Cl, T = transition metal, carbon),^[^ ^122^, ^123^, ^124^, ^125^ ^]^ they get close to the most electron rich region of the halogen (namely, to the lone pairs that form a belt orthogonal to the X–T covalent bond) and the H···X─T angle is close to 90°. If these interactions are correctly named hydrogen bonds,^[^ ^126^ ^]^ the experimental interaction geometry is implied and communicated by their name. The halogen bond^[^ ^40^ ^]^ is the interaction wherein halogen atoms are the electrophile and when these H···X─T interactions have been misnamed halogen bonds,^[^ ^118^ ^]^ this term was wrongly communicating that the H···X─T angle was close to 180° as this is, by definition,^[^ ^40^ ^]^ the geometry around the halogen in HaB.

Conversely, hydrogen atoms bonded to less electronegative atoms (e.g., Z─H, Z═Si, Ge, Sn, Ta, Re, etc.) have an hydridic character, namely, a partial negative charge. When interacting with monovalent and electron poor halogens, these hydrogen atoms function as nucleophiles and get close to the most electron poor region of the halogen, namely, the σ‐hole on the extension of the X─T covalent bond and the H···X─T angle is close to 180°.^[^ ^127^, ^128^, ^129^, ^130^ ^]^ If, following the IUPAC definition,^[^ ^40^ ^]^ these interactions are named halogen bonds,^[^ ^127^, ^128^, ^129^, ^130^, ^131^, ^132^ ^]^ the observed interaction geometry is implied by their name. P. Hobza named these bondings “hydridic hydrogen bonds”,^[^ ^119^, ^120^, ^121^ ^]^ but this wording is an oxymoron as according to the IUPAC definition,^[^ ^52^, ^53^ ^]^ HB is formed by “a hydrogen atom from a molecule or a molecular fragment X─H in which X is more electronegative than H” and thus H has to have a partially positive, not negative, charge. More relevant to the issues considered here, Hobza's terms is not unequivocally communicating that the H···X─T angle in these systems is close to 180° as the opposite polar characters implicit in the term hydridic hydrogen bonds translate into diverse possible H···X─T geometries (this angle should be close to 90° in HB and close to 180° in an hydridic bond).^[^ ^133^ ^]^

### 2.3 Halogen Bond (HaB): the First Tile of a Mosaic

The HaB has been studied and exploited more extensively than the analogous interactions wherein elements of other groups are the electrophile. This interaction will thus be used to tackle a wide array of general issues. In this section and in the two following ones, findings obtained in different fields, understood within different conceptual frameworks and described by using different terminologies, will be presented, adopting a historical perspective to better highlight the gradual development of a multilayered but coherent understanding of the interaction and the gradual development of a consensus on the term halogen bond.

The I_2_···NH_3_ dimer, the first halogen bonded adduct, was prepared in 1813 and its composition established in 1863.^[^ ^134^, ^135^ ^]^ Several other adducts that we now understand as halogen bonded systems had been prepared already in the 19th century, e.g., starting also from Br_2_, Cl_2_,^[^ ^136^ ^]^ and halocarbons.^[^ ^137^ ^]^ Many different terms had been employed for the interactions that drive the formation of these adducts. In his review published in 1968,^[^ ^138^ ^]^ H. A. Bent listed 20 different names or wordings used for interactions wherein halogens and other p‐block elements act as electrophiles. This points to the struggle to developing a consensus on the terminology for related phenomena. Some of these wordings refer to geometric features (bumps‐in‐hollows), some others to chemical features (secondary acid–base interactions), physical phenomena (charge‐transfer), and quantum mechanical aspects (filling of antibonding orbitals).

**Figure 6:**
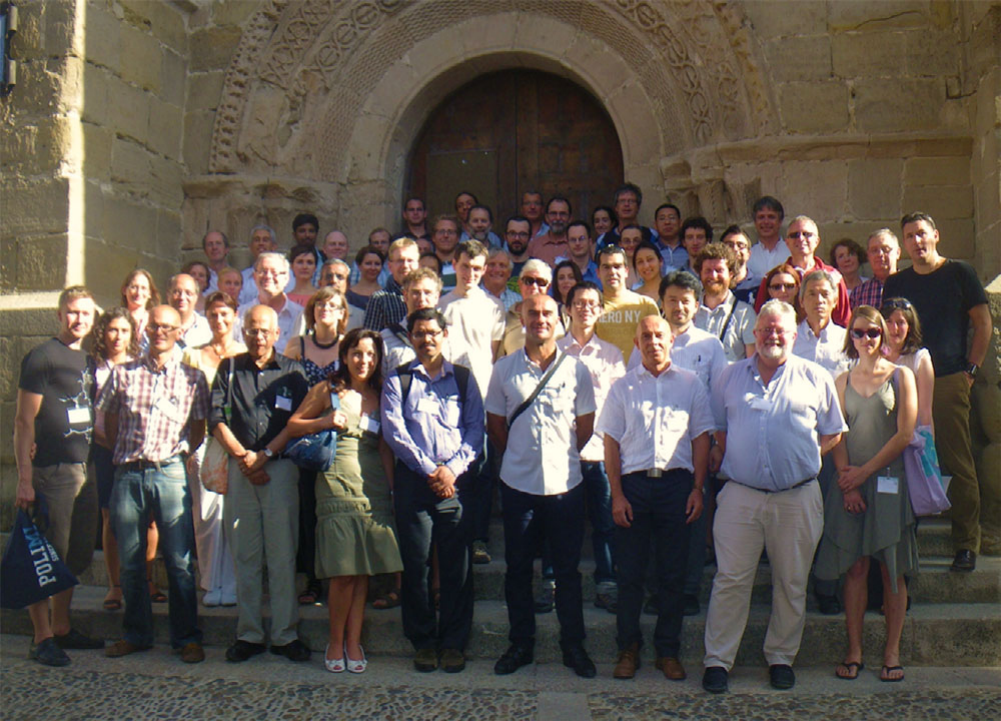
Picture of the participants to the IUPAC workshop in Siguenza where the HaB definition was finalized.

The term “halogen bond” was probably first used for interactions wherein halogen atoms are the electrophile, by R. A. Zingaro in 1961.^[^ ^139^ ^]^ The term was used occasionally in the 1970s^[^ ^140^, ^141^ ^]^ and 80s.^[^ ^142^ ^]^ In the late 90s, the interest in the interaction boomed thanks to the sync of theoretical^[^ ^143^ ^]^ and experimental^[^ ^144^ ^]^ findings and the term halogen bond began to be employed systematically. A symposium on the interaction at the 238th National Meeting of the American Chemical Society (Washington, DC, 16th August, 2009) registered the emerging consensus on understanding within a unified frame a variety of previously disjoined recognition and self‐assembly processes that had been observed in the gas, liquid, and solid phases, and on naming halogen bond the driving interaction. As stated in the highlighting of the event by *Chem. & Eng. News*,^[^ ^145^ ^]^ during the meeting, an agreement developed that the HaB was “noncovalent electrostatic interaction between an electron‐poor Lewis acid and an electron‐rich Lewis base”. Two years later, the understanding of the interaction was perfected during the kick‐off event of an IUPAC project entitled “Categorizing Halogen Bonding and Other Noncovalent Interactions Involving Halogen Atoms”.^[^ ^146^ ^]^ The event was held in Siguenza (20th–21st August, 2011, Spain) (Figure 6) as a satellite workshop of the XXII Congress and General Assembly of the International Union of Crystallography (IUCr2011, Madrid, Spain). The core of the discussion was about the fundamental bonding feature(s) that had to be recalled by the bonding name, the important features to be included in the few words of its definition, and the remaining features that are frequently, but not necessarily present in the bonds.

**Figure 7:**
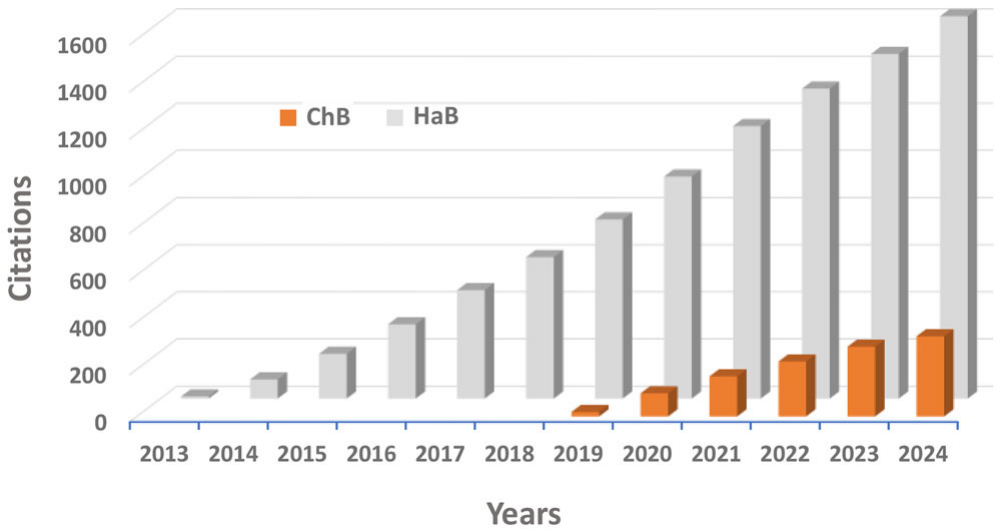
Total number of citations versus years for the IUPAC Recommendations defining the HaB^[^ ^40^ ^]^ and ChB.^[^ ^41^ ^]^ Data (from Scopus) suggest a major interest in the two definitions and interactions.

The consensus needed for finalizing the IUPAC definition^[^ ^40^ ^]^ of the interaction was reached (Figure 7). It was agreed that the elements involved in the interaction formation were the most fundamental and invariant features and had to be recalled by the interaction name. In order to be consistent with the HB, whose name is making a reference to the electrophile, it was decided that the name of the interactions refers to the group 17 of the periodic table. It was also agreed that the electrophilic character of the halogens was the second most important feature of the interactions, whereas the geometric, spectroscopic, and quantum chemical features were useful tools for the reliable characterization of the electrophilicity.

The HaB nature, namely, the origin of the attractive forces allowing for the interaction formation, is a nice example of the inconvenient consequences of naming an interaction by referring to one of its features. The HaB is presently described as a σ‐hole interaction, and this understanding analyzes interactions from the electrostatic point of view, namely, considering their σ‐hole.^[^ ^43^, ^44^, ^45^, ^46^, ^47^, ^48^, ^147^ ^]^ The σ‐hole depth (*V*_s_, max) in X_3_C─I (X = F, Cl, Br, I) increases, moving from X = I to X = F, whereas the HaB strength in X_3_C─I···NMe_3_ and X_3_CI···I^−^ adducts increases moving from X = F to X = I. Clearly, the electrostatic model of the HaB is unable to explain the change in the interactions strength in these systems. Differently, the charge‐transfer model succeeds as calculations show that on changing X, the change in the charge‐transfer component of the interaction is greater than the change in the electrostatic component.^[^ ^148^ ^]^ Some studies on analogous halogen bonded adducts show that electrostatic and dispersion accounts for a half of the overall adducts stability.^[^ ^149^ ^]^ Indeed, many halogen bonded adducts, which are now considered σ‐hole bonded adducts, have been discussed as charge‐transfer and/or donor–acceptor complexes by R. S. Mulliken in his seminal papers.^[^ ^77^, ^78^ ^]^ The same rationalization was typically used by other authors for other similar systems in the 1970s and 80s.^[^ ^138^, ^150^ ^]^

In 1996, in a paper entitled “The nature and geometry of intermolecular interactions between halogens and oxygen or nitrogen”, F. H. Allen stated that “the attractive nature of the interaction is mainly due to electrostatic effects, but polarization, charge‐transfer, and dispersion contributions all play an important role in causing interpenetration of van der Waals volumes”.^[^ ^151^ ^]^ This point of view was very inspiring for filing the fifth HaB feature listed in the IUPAC definition of the HaB,^[^ ^40^ ^]^ which states that “The forces involved in the formation of the halogen bond are primarily electrostatic, but polarization, charge transfer, and dispersion contributions all play an important role. The relative roles of the different forces may vary from one case to the other.” This stance was fully confirmed by researches performed after the definition publication, and in 2021, S. Kozuch concluded with some irony that HaB, as well as other similar interactions, are “an amalgamation of several key effects: electrostatic, polarization, dispersion, and charge‐transfer. The weight of each one of these components depends on the type of moieties in the adduct, on the bond distance, on the method used to quantify the effects, and some would even say that it also depends on the researcher's whim.”^[^ ^152^ ^]^

Halogen atoms were described as the Lewis acidic atoms in the HaB at the 238th ACS National Meeting^[^ ^145^ ^]^ and as the electrophilic atoms in the IUPAC definition.^[^ ^40^ ^]^ Although Lewis acidity and electrophilicity are related concepts and largely interchangeable terms (and the same holds for Lewis basicity and nucleophilicity), the former is a thermodynamic parameter and the latter a kinetic one (“Lewis acidity is measured by relative equilibrium constants, and electrophilicity is measured by relative rate constants for reactions of different electrophilic reagents toward a common substrate”).^[^ ^153^ ^]^ It seems that the choice of the IUPAC recommendation to define the HaB as an electrophile/nucleophile interaction rather than a Lewis acid/Lewis base interaction was very convenient and farsighted. It is now well established that halogen atoms that have all the features of Lewis bases, when considered as isolated molecular entities in the ground state, can act as electrophiles and form attractive HaBs. This is the case for halogen atoms that, in a neutral molecule or in an anion (e.g., bromine or iodine in BrO_3_^¯^, IO_3_^¯^, or IO_4_^¯^ anions), have a negative electrostatic potential on their whole surface, i.e., a negative σ‐hole.^[^ ^154^, ^155^ ^]^ But in the solid or in solution, the electron density distribution on the halogen changes as a result of the polarization induced by the interactions with the surrounding molecular entities. The negative electrostatic potential at some regions turns to positive and these regions attractively interact with negative regions of nearby molecular entities. HaBs are formed in which both the electrostatic and the charge‐transfer components of the interaction contribute to the stability of the formed short contacts. The changes of the electron density distribution that occur due to polarization are better acknowledged when using electrophilicity, with its kinetic roots, as the distinctive feature of the HaB, than when using Lewis acidity, with its thermodynamic basis.

The great attention to the basic aspects of the HaB and the discussion on its naming were a result of the relevance of the interaction and examples of useful applications are given below.

The HaB affects/drives the binding of small molecules to biomacromolecules and determines their conformation, folding, and aggregation.^[^ ^156^ ^]^ In 2003, P. S. Ho reported a four‐stranded DNA Holliday junction stabilized by a Br···O HaB between the bromine of a 5‐bromouracil unit and the oxygen of a phosphoric unit.^[^ ^157^ ^]^ One year later the same author with P. Auffinger established, via a survey of protein and nucleic acid structures, the general ability of inter‐ and intramolecular HaBs to affect ligand binding and biomacromolecular folding.^[^ ^158^, ^159^ ^]^ The biological relevance of the HaB encompasses synthetic haloorganics and naturally occurring ones. Thyroxine, a prohormone, forms short I···O HaBs with transthyretin, its transport protein.^[^ ^160^ ^]^

**Figure 8:**
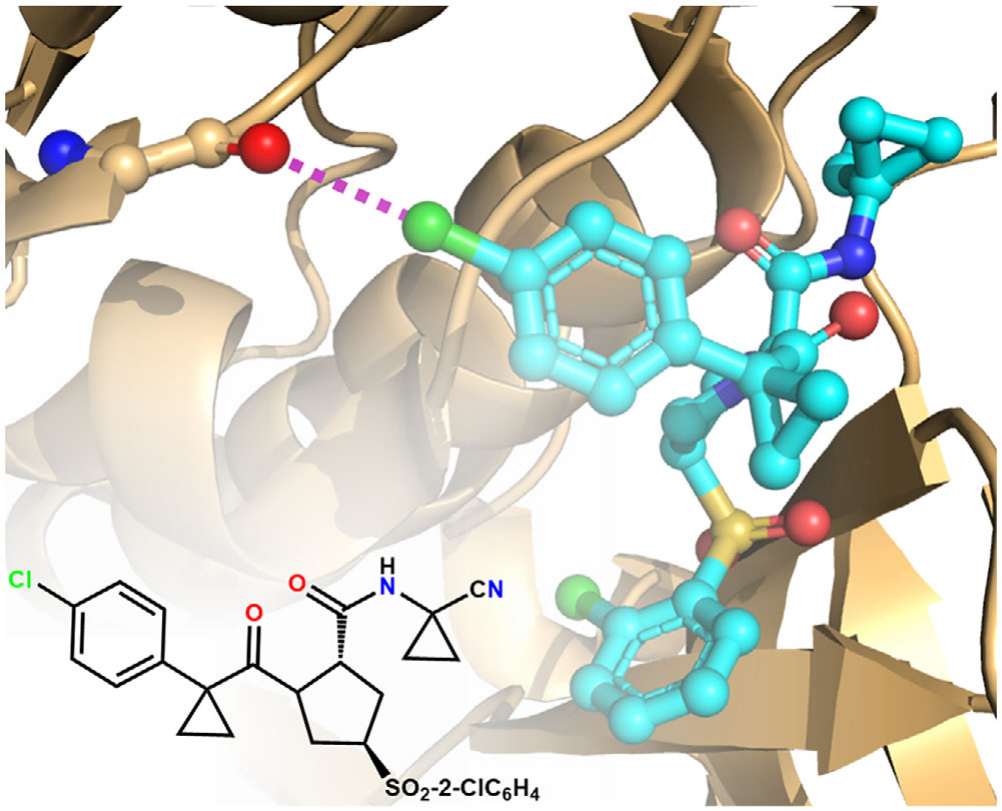
Partial view of the complex (PDB code 2XU1) between hCatL and an inhibitor carrying a 4‐chlorophenyl moieties.^[^ ^169^ ^]^ The structural formula of the inhibitor is in the bottom‐left corner, the Cl····O HaB is the violet dotted line.

HaB has been used in a wide diversity of biooriented application, spanning the amplification of amyloid self‐assembly,^[^ ^161^ ^]^ the increase of membrane association, permeability or penetration,^[^ ^162^, ^163^, ^164^, ^165^, ^166^, ^167^, ^168^ ^]^ and drugs optimization. For example, the dysregulated function of human cathepsin L (hCatL) is involved in a series of human diseases. The discovery of novel and more effective hCatL inhibitors typically looks for the substituents on lead scaffolds that maximize the binding affinity. The IC_50_ values of some hCatL inhibitors, wherein a 4‐halophenyl residue is appended to a proline,^[^ ^169^ ^]^ increase when the halogen changes from F to I (F << Cl < Br << I), namely, IC_50_ values increase with the HaB donor ability, consistent with a role of the HaB in the drug/protein binding. Such a role is confirmed by the crystal structure of the complex between hCatL and the 4‐chlorophenyl substituted inhibitor that displays a Cl···O HaB between the Cl of the phenyl ring and the O of Gly61 in the S3 pocket (Figure 8).

**Figure 9:**
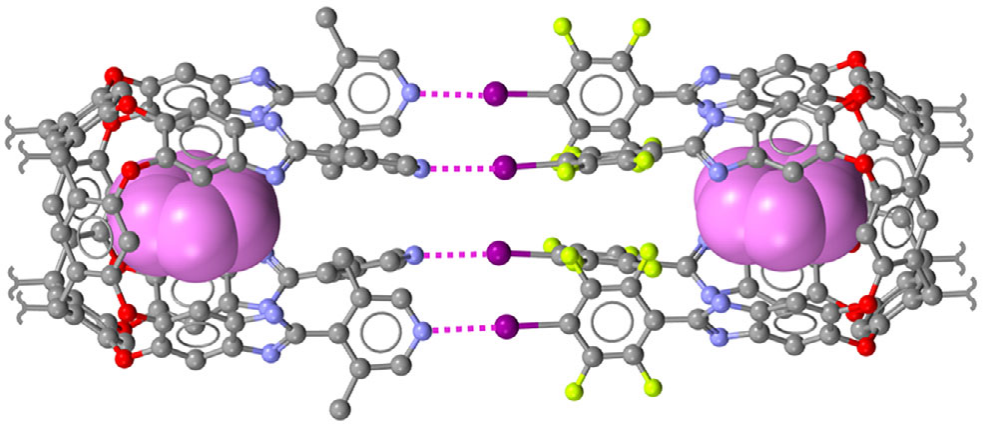
Representation of the capsule (single‐crystal X‐ray structure) assembled via I···N HaBs (purple dotted lines) between lutidine and iodotetrafluorophenyl moieties appended to resorcinarene scaffolds.^[^ ^172^ ^]^ Encapsulated benzene molecules are in spacefilling mode and pink color; the alkyl pendants at resorcinarene moieties have been deleted. Color code: carbon, grey and pink; nitrogen, indigo; oxygen, red; iodine, purple; fluorine, yellowish green.

Crystal engineering is another field of successful exploitation of the HaB.^[^ ^170^ ^]^ The interaction allowed to design and obtain useful systems; for example, supramolecular containers.^[^ ^171^ ^]^ Two resorcin[4]arene cavitand scaffolds functionalized with tetrafluoroiodophenyl motifs (HaB donors) and lutidyl pendants (HaB acceptors) pair into dimers that are stable in the solid state^[^ ^172^ ^]^ (Figure 9), in solution, and in the gas phase.^[^ ^173^ ^]^

**Figure 10:**
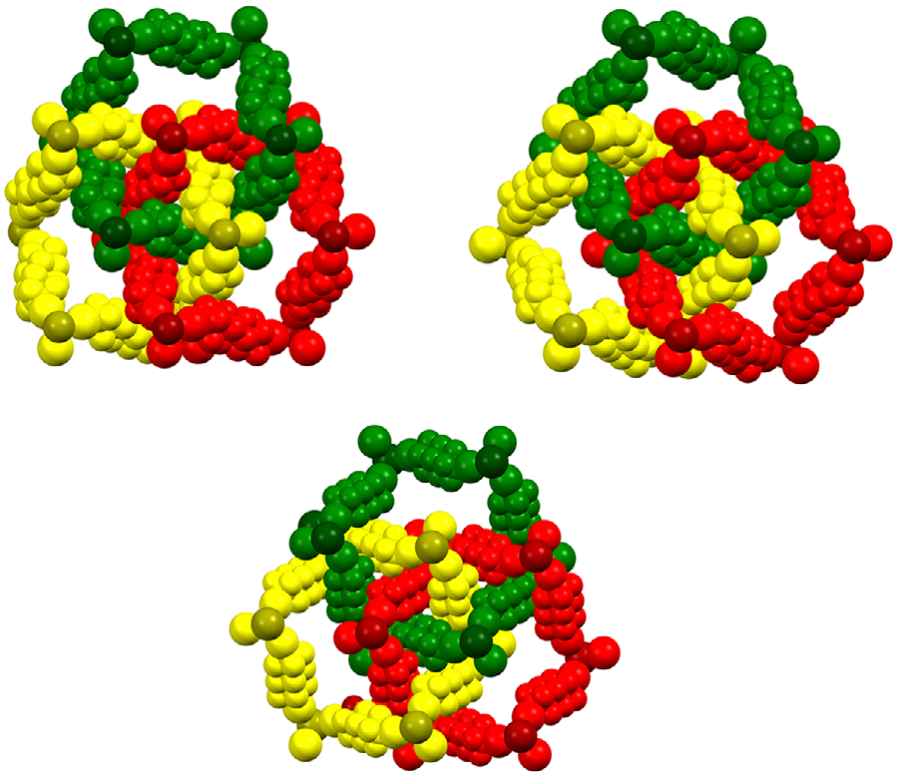
Space filling representation of three rings of the Borromean interpenetrated layers present in crystals formed by KCl/K‐2.2.2/I–(CF_2_)_8_–I (top left), RbBr/K‐2.2.2/I–(CF_2_)_8_–I (top right), and NH_4_I/K‐2.2.2/I–(CF_2_)_6_–I (bottom).^[^ ^174^ ^]^ The cryptated cation is not reported; the three rings are depicted with different colors, the halide anions are darker than the iodoperfluorocarbon chains they are bonded to.

The versatility of HaB allowed for obtaining topologies as sophisticated as layers with Borromean interpenetration. A small library of Borromean interpenetrated and supramolecular structures is obtained when halide salts, the [2.2.2]‐cryptand, and α,ω‐diiodoperfluoroalkanes self‐assemble in solutions^[^ ^174^ ^]^ or via solvent‐free mechanosynthesis.^[^ ^175^ ^]^ The reliability of the HaB in controlling the self‐assembly of these three compounds’, four components’ systems is confirmed by the fact that Borromean interpenetrated nets are formed starting from sodium, potassium, rubidium, and ammonium cations, from chloride, bromide, and iodide anions, from 1,6‐didiodoperfluorhexane and 1,8‐diiodoperfluorooctane (Figure 10).

**Figure 11:**
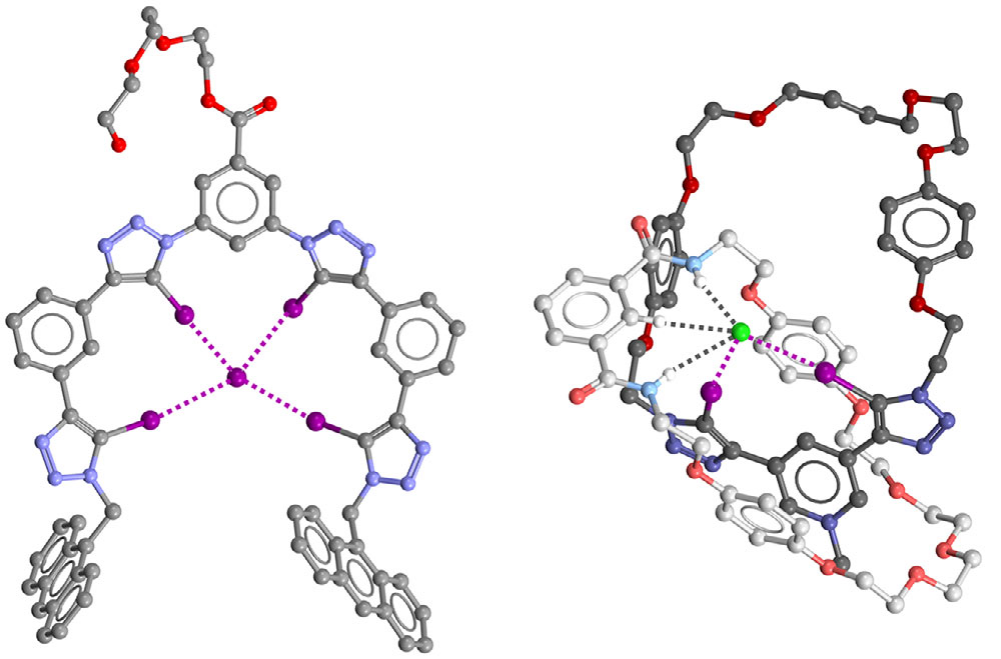
Representation of the single‐crystal X‐ray structure of: neutral foldamers functioning as tetradentate HaB receptor of I^− [^ ^178^ ^]^ (left); [2]catenane binding Cl^−^ in an interlocked 3D cavity; atoms in one ring are darker than in the other.^[^ ^179^ ^]^ Hydrogens are omitted but those involved in HB formation; color code: carbon, grey; hydrogen, whitish; nitrogen, indigo; oxygen, red; iodine, purple; chlorine, green.

Anion recognition is an important topic, mainly due to its strong implications in areas as different as medical diagnostics and detection/sequestration of toxic (CN^−^, AsO_4_^2−^) or environmentally impacting anions (NO_3_^−^, PO_4_^3−^).^[^ ^176^ ^]^ The tendency of many anions to function as effective HaB acceptors led to the development of a variety of HaB based anion receptors^[^ ^177^ ^]^ that often display higher anion binding constant than their HB based analogues. P. D. Beer reported neutral foldamers functioning as tetradentate HaB anion receptors thanks to the presence of four 5‐iodo‐1,2,3‐triazole moieties (Figure 11, left).^[^ ^178^ ^]^ These foldamers show stronger affinity for halides (especially I^−^ and Br^−^) and oxoanions than the analogous HB based systems.

The same author reported systems where a bis‐iodotriazole‐pyridinium motif was included in acyclic and [2]catenane anion host systems (Figure 11, right).^[^ ^179^ ^]^ The acyclic receptor displays selectivity for acetate over halides and improved anion recognition abilities compared to the analogous HB based acyclic receptor. Catenane receptors show high selectivity for halide anions even in competitive organic–aqueous solvent mixtures.

The effectiveness of anions as HaB acceptors led rapidly to the development of HaB based systems for anions transport across lipid bilayer membranes.^[^ ^162^ ^]^ Synthetic anion transporters relying on HaB present some advantaged with respect to systems based on HB. First, they have lower solvation energies than transporters with polar HB groups. Second, HaB is more directional than HB and selective binding can be more easily obtained. Third, binding strength can be tuned by changing the halogen and the nearby residues. Fourth, and possibly most important, halogenated moieties cannot be easily protonated/deprotonated, in contrast to HB based transporters that contain OH or NH groups, and consequently they cannot lead to pH changes and cause in vivo toxicity. S. Matile first proved the role of HaB in anion transport^[^ ^180^ ^]^ and showed that HaB donors as simple as 1‐iodoperfluoroalkanes act as selective, leakage free, cooperative, and nonohmic transporters.^[^ ^168^ ^]^

### 2.4 Chalcogen and Pnictogen Bonds: Two Other Tiles of the Mosaic

Similar to the HaB, the single experimental findings and theoretical results presently encompassed by the terms chalcogen bond^[^ ^41^ ^]^ and pnictogen bond^[^ ^42^ ^]^ have been obtained in studies performed in quite different contexts over a wide span of time. Pieces of information has long remained fragmented and understood within different conceptual frames long before the common features were recognized. This unification occurred at the beginning of the 21st century as a corollary of the HaB.

To the best of our knowledge, the first report on chalcogen bonded cocrystals dates back to 1843 when A. W. Hofmann described the SO_2_/amines adducts.^[^ ^181^ ^]^ As now proven by crystallographic^[^ ^182^, ^183^ ^]^ and computational^[^ ^184^ ^]^ results, these complexes are assembled under control of the attractive interactions between nucleophiles and the region of depleted electron density that is present above and below the chalcogen atom. Analogous complexes wherein phenols and anilines substitute for amines were reported in 1882^[^ ^185^ ^]^ and 1891.^[^ ^186^ ^]^

**Figure 12:**
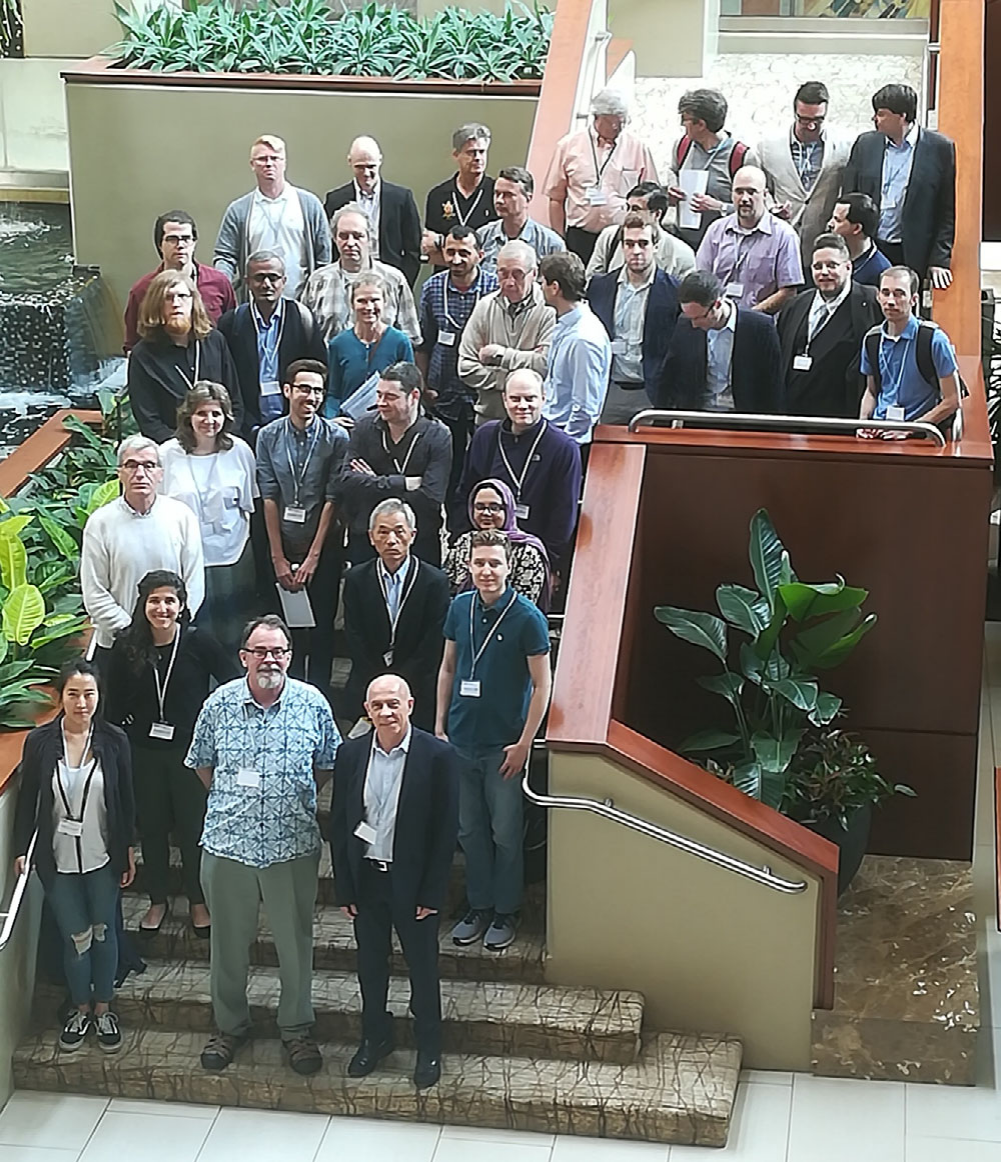
Participants of the IUPAC workshop in Greenville where a consensus on the main aspects of the ChB definition was reached (with copyright permission from Elsevier).

The tendency of divalent sulfur atoms forming two σ covalent bonds to give interactions now understood as σ‐hole ChBs (namely, the attractive interactions wherein nucleophiles get close to a chalcogen atom along the extension of a covalent bonds involving the chalcogen) was first recognized by R. Parthasarathy in 1977^[^ ^187^ ^]^ through an analysis of the Cambridge Structural Database (CSD). But the potential of this finding remained unexploited until the beginning of the 21st century when J. S. Murray proposed to rationalize these interactions, and the analogous ones formed by selenium, in terms of the attraction between nucleophiles and one of the σ‐holes (i.e., the regions of depleted electron density and usually with positive electrostatic potential) that is present on the outer surfaces of chalcogen atoms.^[^ ^188^ ^]^ This paper attracted on the topic the attention of researchers involved in different fields and rapidly a flurry of papers appeared proving the usefulness of self‐assembly and recognition processes driven by these interactions. The need for a consistent and unambiguous nomenclature for these bondings became immediately evident as, similar to the HaB, alike interactions formed by electrophilic elements of group 16 were named differently by different authors and/or in different contexts.^[^ ^105^, ^106^, ^107^, ^108^, ^138^ ^]^ This terminological problem was tackled by an IUPAC project entitled “Categorizing Chalcogen, Pnictogen, and Tetrel Bonds, and Other Interactions Involving Groups 14–16 Elements”. The researchers participating to the kick‐off event of the project were from Europe, North America, and Asia and they agreed that it was convenient to pursue “a systematic and periodic terminology naming any interaction wherein an electrophilic partner can be identified, from the Group of the Periodic Table to which the electrophilic atom belongs”.^[^ ^189^ ^]^ A consensus was reached that the interactions wherein the group 16 elements are the electrophile should be named chalcogen bonds (ChBs), and that, for the sake of systematicity, the IUPAC definition of this term should parallel the IUPAC definitions of HB and HaB. The manuscript for the IUPAC definition of the ChBs was finalized during an international workshop entitled “Interactions Involving Group 14–16 Elements as Electrophilic Sites: A World Parallel to Halogen Bond” which was organized in Greenville (9th–10th June, 2018, SC, USA) as a satellite event of the Third International Symposium of Halogen Bonding (ISXB‐3) (Figure 12). Importantly, during that workshop it was agreed that the term chalcogen bond had to be used for interactions where the nucleophile is getting close to the chalcogen both on the extension of a σ covalent bond formed by the chalcogen and orthogonal to a chalcogen atom adopting a planar geometry and forming a π bond (namely, for the interactions that are now commonly named both σ‐hole and π‐hole bonds). Moreover, it was considered that, in order to designate the chalcogen bond, the three letters acronym ChB had to be preferred over the two letters acronym CB, which had been used for weak bonds wherein carbon is the electrophile and confusion may arise.^[^ ^83^, ^84^, ^85^ ^]^

**Figure 13:**
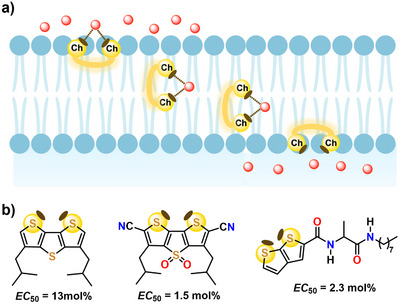
a) Schematic representation of chalcogen bond–mediated chloride anion (red balls) transportation across bilayer lipid membrane via a bidentate system. Brown ellipsoids around chalcogen atom represent σ‐holes. b) Some ChB‐based and sulfur‐containing anion transporters with EC_50_ values (in mol%) for Cl⁻.^[^ ^190^ ^]^

The ChBs has proven its usefulness in several fields and here we report some interesting applications. The directionality of the interaction and the possibility to tune its strength by changing the electron withdrawing ability of residues close to the chalcogen were used by S. Matile to obtain effective anion transport across lipid bilayers.^[^ ^190^ ^]^ The rigid and planar geometry of the electron‐deficient dithieno[3,2‐b;2′,3′‐*d*]thiophene moiety allowed for the preorganization of two σ‐holes on two different sulfur atoms for the convergent binding of a single anion (Figure 13a,b). Oxidation of the sulfur not involved in the anion binding increases the binding and the EC_50_ value for Cl⁻ transport improves from 13 to 1.5 mol%. Also, the thieno[3,2‐*b*]thiophene moiety allows for effective anion transport. When bearing a hydrocarbon pendant, this system self‐assembles into transport channels showing a transport activity that is high for Cl⁻ (EC_50 _= 2.3 mol%) and very high for ClO₄⁻ (EC_50 _= 0.3 mol%).^[^ ^191^ ^]^

**Figure 14:**
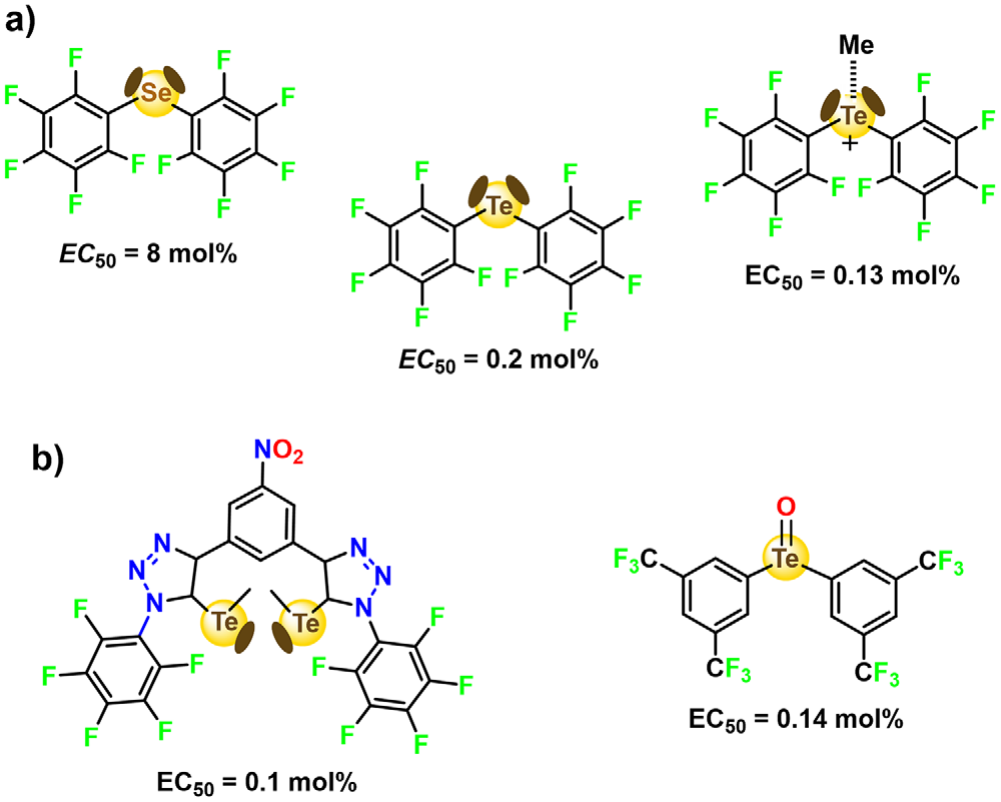
a) Bis(pentafluorophenyl)selenide and telluride derivatives and b) structurally more complex organic tellurides acting as anion transporter; EC_50_ values for Cl⁻ are given in mol%.^[^ ^192^, ^193^, ^194^, ^195^ ^]^

Other chalcogens can similarly enable for effective anion transport. Bis(pentafluorophenyl)telluride and its selenium analogue are effective Cl⁻ transporters (Figure 14a), the former compound outperforming the latter.^[^ ^192^ ^]^ F. P. Gabbaï reported that the telluronium cation obtained by methylation of bis(pentafluorophenyl)telluride performs even slightly better in Cl⁻ transport.^[^ ^193^ ^]^

The effective Cl⁻ transport shown by a bis(telluromethyltriazole) system may be related to the preorganization of the two chalcogens toward a convergent anion binding (Figure 14b).^[^ ^194^ ^]^ A diaryltelluroxide shows a similar Cl⁻ transport activity and offers the possibility to switch on and off the transport via in situ oxidation and reduction of tellurium.^[^ ^195^ ^]^ This small inventory of structures proves that Te based anion transporters outdo in activity sulfur and selenium analogues.

**Figure 15:**
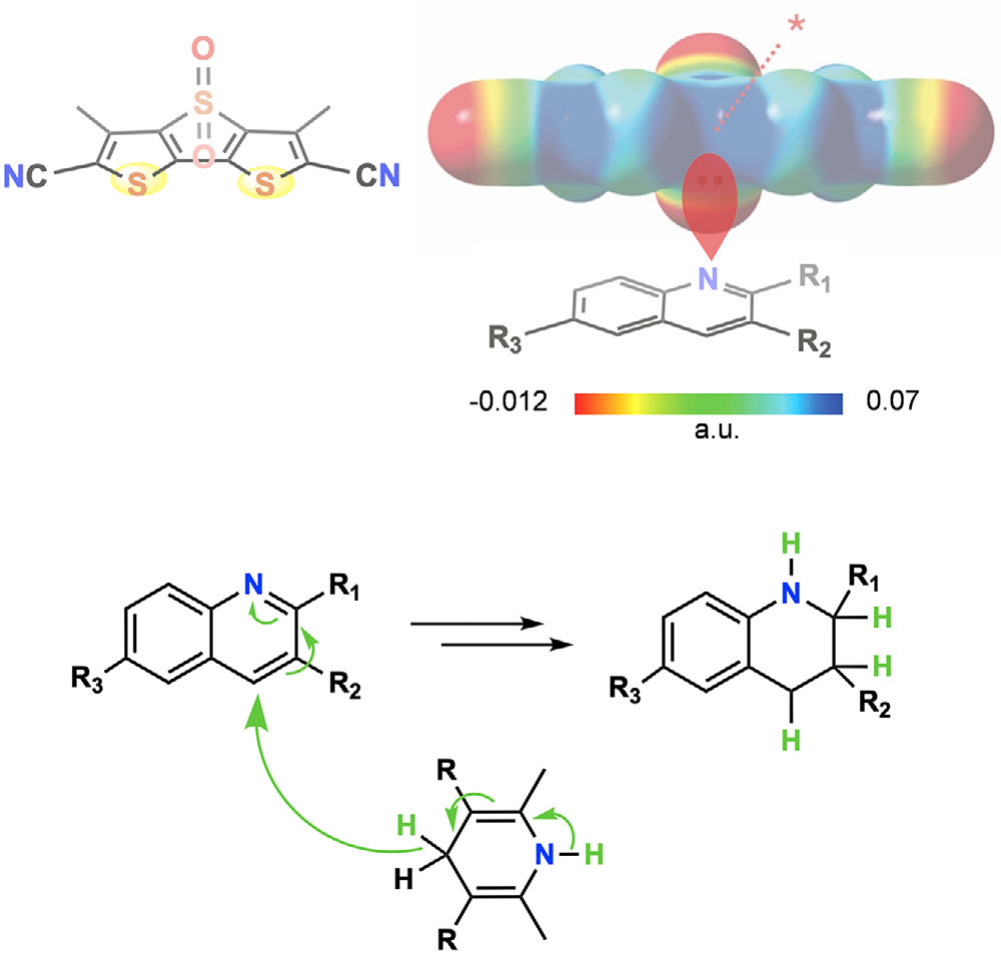
Top: structural formula of the dithienothiophene *S*,*S*‐dioxide used as catalyst and its molecular electrostatic potential surface (MEP, at MP2/6‐311++G**//M062X/6‐311G**, isosurface: 0.009 au; red: −0.012 a.u., blue: 0.07 a.u.); the point of coalescence of iso‐oriented sulfur σ‐holes in indicated by the red dotted line and star. Bottom: scheme of the ChB catalyzed transfer hydrogenation reactions between dihydropyridines and quinolines.^[^ ^196^, ^197^ ^]^

The directionality and hydrophobicity of the ChB are a plus also when the interaction is used in organo‐catalyses. Seminal results in this field were reported by S. Matile who leveraged the anion transport ability of dithienothiophene based systems in the transfer hydrogenation reactions of quinolines and imines (Figure 15).^[^ ^196^, ^197^ ^]^ The double pinning of the lone pair of quinoline nitrogen by the two iso‐oriented sulfur atoms of the catalyst boosts the electrophilicity of quinoline para position and activates the transfer of the hydride, and then of the proton, from the dihydropyridine reagent.

V. Mamane demonstrated impressive catalytic properties of telluronium cations in several benchmark reactions spanning the aza‐Diels–Alder between Danishefsky's diene and imines, the bromolactonization of ω‐unsaturated carboxylic acids, and the Friedel–Crafts bromination of anisole.^[^ ^198^ ^]^

**Figure 16:**
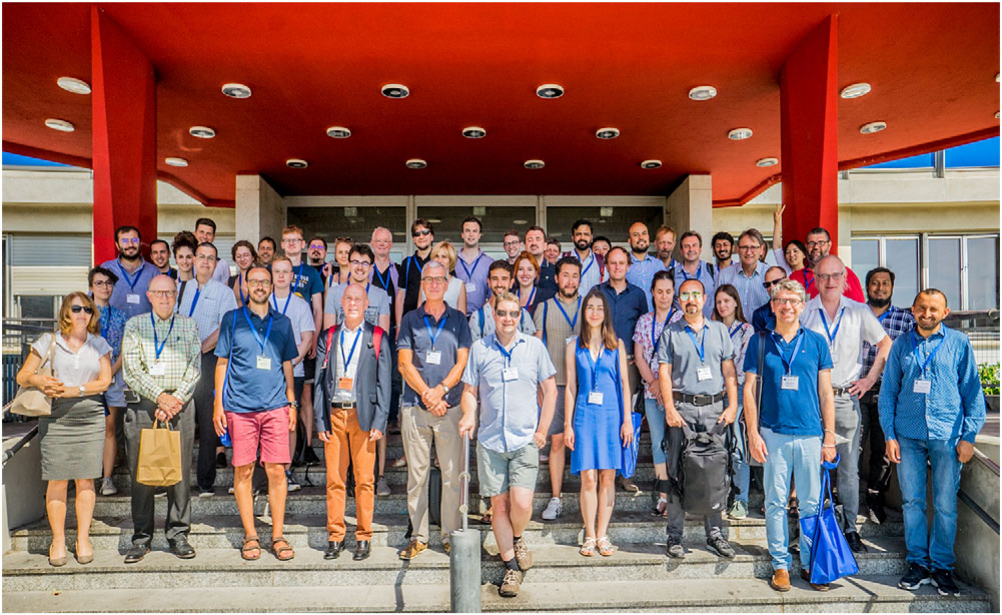
Social picture of the participants to the IUPAC workshop in Strasbourg where the specific features of PnB were discussed and the fundamental aspects of the PnB definition identified.

Interactions, wherein group 15 elements are the electrophile, present similarities and differences with those formed by electrophilic elements of group 17 and 16. Similar to the HaB and ChB, the naming of the weak interactions wherein pnictogens are the electrophile had to cope with the challenge to organize in a single and unified model experimental and theoretical findings obtained within different fields, analyzed via different techniques, and understood by employing different conceptual frames. But there were also the additional challenges related to the greater diversity in the structural and interactional aspects of pnictogen derivatives with respect to the halogen and chalcogen derivatives. Importantly, the heavier pnictogen elements have a pronounced metallic character, greater than that of chalcogen and halogen elements of the same period. It is not uncommon that pnictogen derivatives form with nucleophiles complexes via interactions whose distances and strengths are similar to the covalent bonds in the uncomplexed species and these interactions are typically understood as coordination bonds.^[^ ^199^ ^]^ These issues were discussed during an IUPAC Workshop organized as a Satellite Event of the 2nd International Conference on Noncovalent Interactions (Strasbourg, France, 2022) (Figure 16). It was agreed to pursue that the PnB definition acknowledges not only the distinctive features of the interaction but also the similarities with the HaB and ChB. Importantly, the use of the term pnictogen bond was limited to the bondings that are substantially longer and weaker than the bonds present in the uncomplexed species.

**Figure 17:**
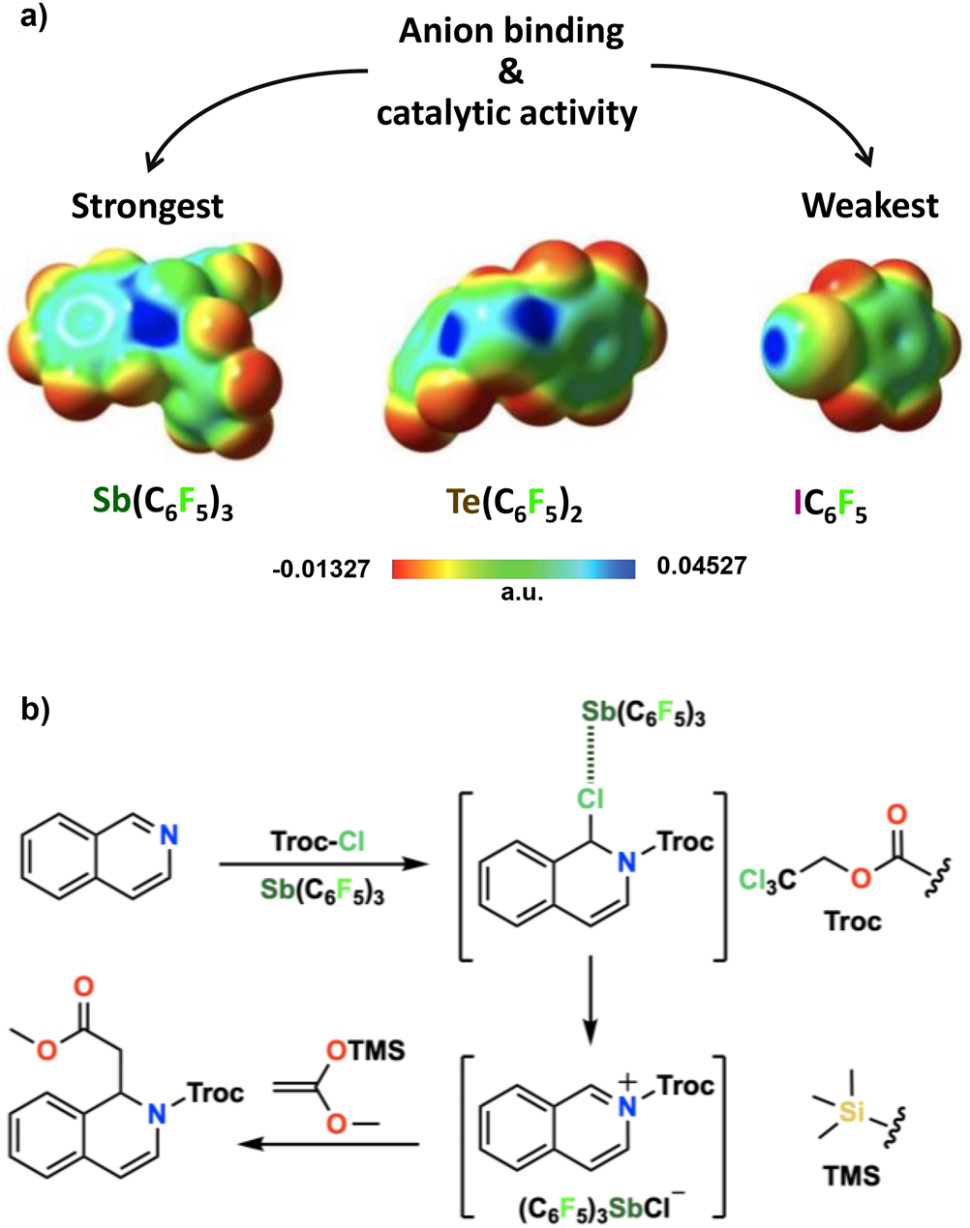
a) MEP surfaces (M06‐2X/6‐311G**/aug_cc‐pVTZ‐pp, isosurface 0.001 a.u.; red: −0.01327 a.u., blue: 0.04527 a.u.) of three pentafluorophenyl substituted derivatives used to catalyze b) a carbon nucleophile substitution for chlorine in a 1‐chloro‐dihydro‐isoquinoline derivative.^[^ ^200^ ^]^

The strive for a correct naming of the interactions wherein group 15 elements are the electrophile were concomitant with the use of these interactions in valuable applications. (C_6_F_5_)_3_Pn (Pn = As, Sb), (C_6_F_5_)_2_Ch (Ch = Se, Te), and C_6_F_5_Ha (Ha = Br, I) as well as some analogous derivatives wherein C_6_H_5_ residues substitute for C_6_F_5_ ones were used for a direct comparison of the effectiveness in catalysis and anion transport of PnB, ChB, and HaB. The reaction of nucleophile for chlorine substitution on dihidroisoquinoline derivatives was employed to compare the activity of the different catalysts (Figure 17). An activity increase was observed moving from group 17 to group 15, from period 3 to period 5, and on increasing the number of C_6_F_5_ residues with respect to C_6_H_5_ ones.^[^ ^200^ ^]^ The trends of used catalysts in the binding of anions (employed as prototype nucleophiles) were established both theoretically and experimentally and varied with the same trend.

Consistent with the Goldilocks principle, which in relation to anion transport may be expresses saying that the strongest anion binders are not the best anion transporters,^[^ ^201^, ^202^ ^]^ the ChB based species were the best Cl⁻ transporters.^[^ ^192^ ^]^ They showed a nanomolar activity and were three order of magnitudes better performers than the HaB based analogues. Their Cl⁻/Na^+^ and Cl⁻/NO_3_⁻ selectivities (*P*_Cl⁻/Na+_ = 10.4 and *P*_Cl⁻/NO3⁻_ = 4.5) were higher than those of structurally similar PnB based derivatives (*P*_Cl⁻/Na+_ = 2.1 and *P*_Cl⁻/NO3⁻_ = 2.5). This was probably due to the fact that PnB transporters were forming membrane‐disruptive supramolecular amphiphiles due to “too strong” binding of anions.

**Figure 18:**
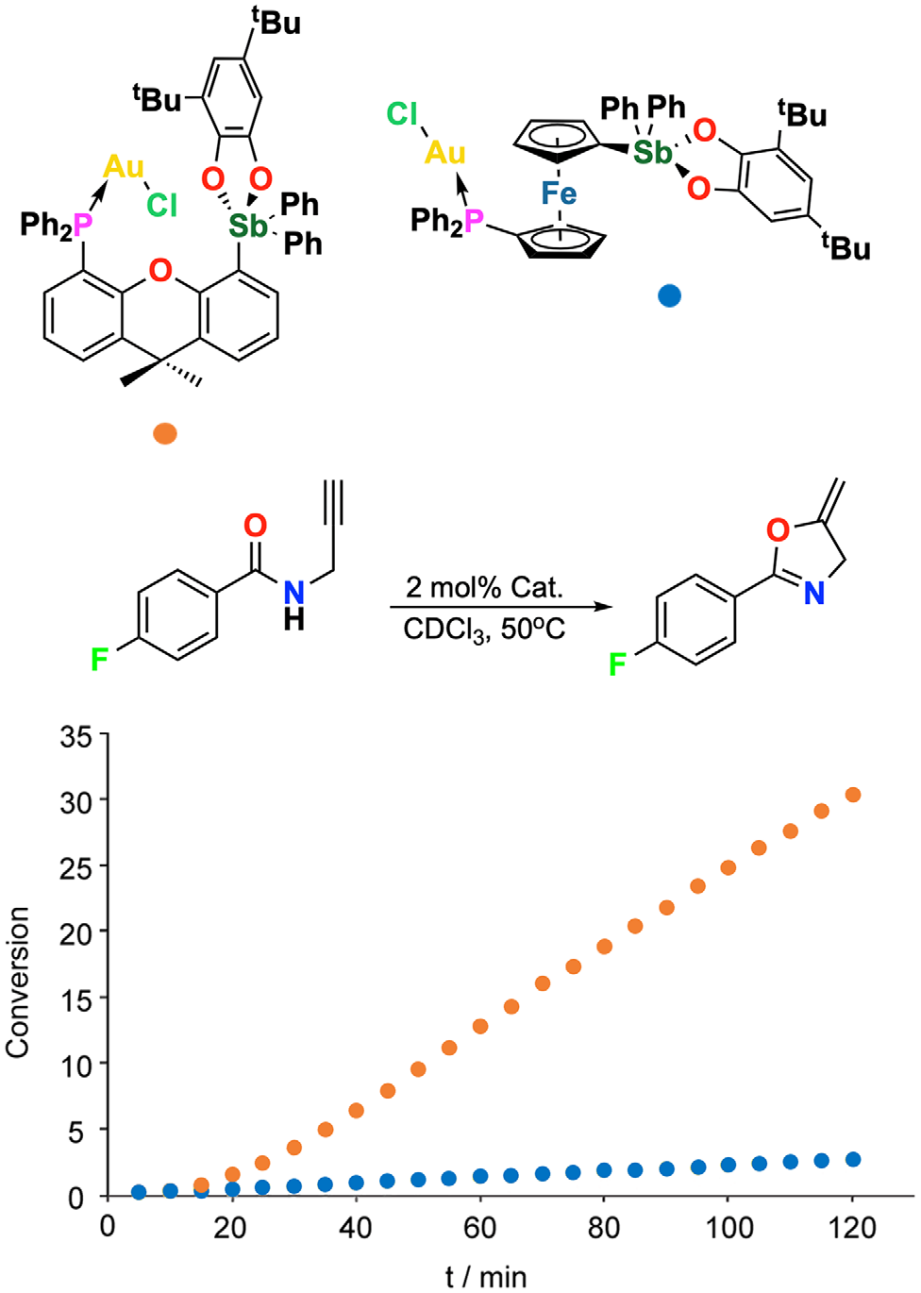
Catalytic effect of the intramolecular Au─Cl→Sb(V) PnB in a gold(I)chloride complex promotes the cycloisomerization of a propargyl amide.^[^ ^203^ ^]^

Another example of PnB effectiveness in catalysis is given by the ability of an intramolecular Au–Cl→Sb(V) interaction to activate a phosphine gold(I)chloride complex in catalyzing the cycloisomerization of a propargyl amide (Figure 18).^[^ ^203^ ^]^

### 2.5 Interactions Wherein Elements of the p and d Blocks Act as Electrophiles

The IUPAC workshop in Strasbourg was entitled “Interactions involving elements of groups 11, 14, 15, 16 and beyond” due to the increasing number of groups of the periodic table whose elements are recognized to form weak bonds presenting several similarities to the interactions described in Sections 2.3 and 2.4. These new entries will be discussed in this section.

Similar to the HaB, ChB, and PnB, the ability of group 14 elements to act as electrophiles/Lewis acids had been recognized and investigated long before the weaker bondings formed by these elements with nucleophiles were understood within the frame of the σ/π‐hole bond modeling and were named with the umbrella term tetrel bonds (TtBs). The Lewis acidity of organic derivatives of germanium, tin, and lead is a well‐developed topic within the chemistry of main group elements.^[^ ^204^ ^]^ The group 14 elements are softer than the groups 15, 16, and 17 elements of the same period and they have greater metallic characters, greater polarizabilities, and lower electronegativities. For these reasons, and some others, e.g., the level of LUMO orbital, complexes with nucleophiles/Lewis bases are more frequently formed via bonds similar in length and strength to the bonds present in uncomplexed parent compounds. These systems have been typically rationalized as coordination or hypervalent complexes.^[^ ^205^, ^206^, ^207^ ^]^ But in some other cases the formed bonds are substantially longer than bonds present in uncomplexed parent compounds and marginally shorter than the sum of van der Waals radii of the involved elements. These contacts can be and have been understood within the frame of σ‐hole bond modeling and have been named TtBs.^[^ ^208^, ^209^, ^210^, ^211^ ^]^

**Figure 19:**
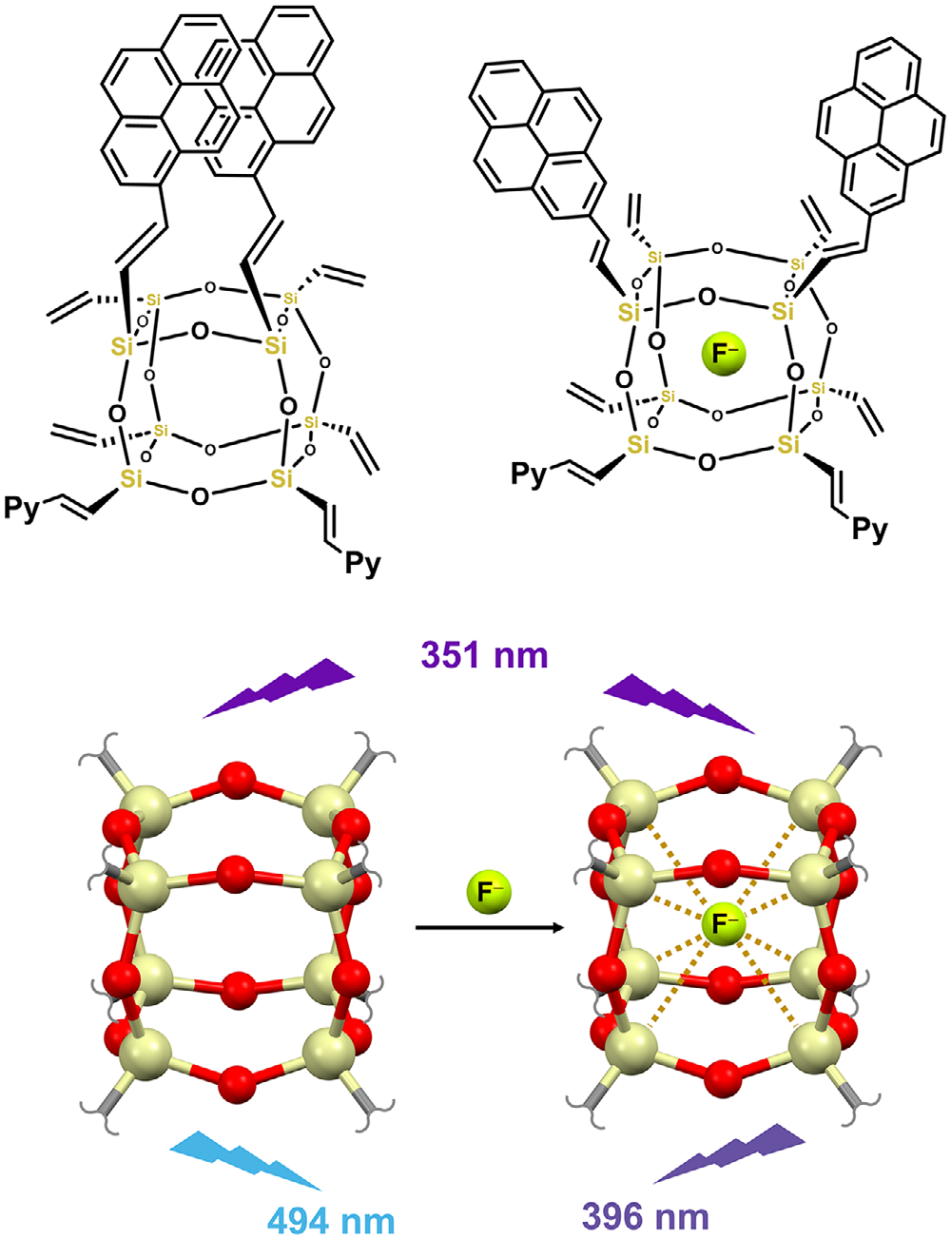
Top: structural formulas of a pyrene functionalized silsesquioxane, which on F^−^ caging changes conformation and photophysical properties. Bottom: ball‐and‐stick representation of a silsesquioxane from the CSD (Refcode IVEPUV) evidencing the trapping of fluoride anions via multiple TtBs (ocher dotted lines); emission properties with and without F^−^ caging are indicated.^[^ ^214^ ^]^

The nonminor difference in the softness, metallic character, polarizability, and electronegativity of silicon with respect to germanium, tin, and lead translates in the more frequent formation of weak Si···nucleophiles bonds characterized by a length close to the sum of van der Waals radii of involved atoms. Interestingly, the complexes between SiF_4_ and amines were the first adducts of a group 14 element rationalized within the frame of σ‐hole bondings^[^ ^212^ ^]^ and the term tetrel bond was first used in a paper focused on the tendency of silicon derivatives to bind anions.^[^ ^57^ ^]^ Thanks to its reliability,^[^ ^213^ ^]^ this tendency has found useful applications in the fluorescent sensing of F^−^ via molecular^[^ ^214^ ^]^ and polymeric^[^ ^215^ ^]^ silsesquioxanes (Figure 19).

Carbon, the hardest, most electronegative, and least metallic and polarizable element of group 14 exceptionally forms, with nucleophiles, short and strong interactions.^[^ ^216^, ^217^ ^]^ The vast majority of interactions between C and nucleophiles are fairly weak and their separations are close to the sum of van der Waals radii of the interacting partners, namely, they perfectly match the typical features of TtBs.

The ability of carbon electrophilicity to drive the formation of weak bonds was first recognized for C(sp^2^) sites, when in 1973, H. B. Bürgi and J. D. Dunitz uncovered, via CSD analyzes, the nearly orthogonal trajectory of approach of nucleophiles to carbonyl carbon atoms.^[^ ^218^, ^219^ ^]^ In 1991, P. Politzer analyzed these contacts in terms of electrostatics^[^ ^220^ ^]^ and in 2012, he rationalized them, and their silyl analogues, within the π‐hole bonds model.^[^ ^184^ ^]^ Now these interactions, as well as the analogous bondings formed by C═N and C≡N moieties, are also named π‐hole TtBs.^[^ ^50^ ^]^ Interestingly, several complexes formed in the gas phase by CO_2_ with nucleophiles (e.g., with acetonitrile^[^ ^221^ ^]^ and benzaldehyde^[^ ^222^ ^]^) have been rationalized as π‐hole tetrel bonded adducts and this binding mode may receive increasing attention in the future in relation to CO_2_ capture and storage.

The presence of σ‐holes on C(sp^3^) atoms is a much more recent story as it was first described in 2009.^[^ ^223^ ^]^ The topic rapidly attracted major interest and a flurry of theoretical studies,^[^ ^83^, ^85^, ^224^, ^225^, ^226^ ^]^ CSD and Protein Data Bank (PDB) searches,^[^ ^56^, ^57^, ^84^, ^227^, ^228^, ^229^ ^]^ and experimental investigations^[^ ^230^, ^231^, ^232^, ^233^ ^]^ has consistently proven the presence of σ‐hole TtBs involving carbon in solution,^[^ ^232^ ^]^in gas,^[^ ^231^ ^]^ and in solid phases.^[^ ^230^, ^233^ ^]^

**Figure 20:**
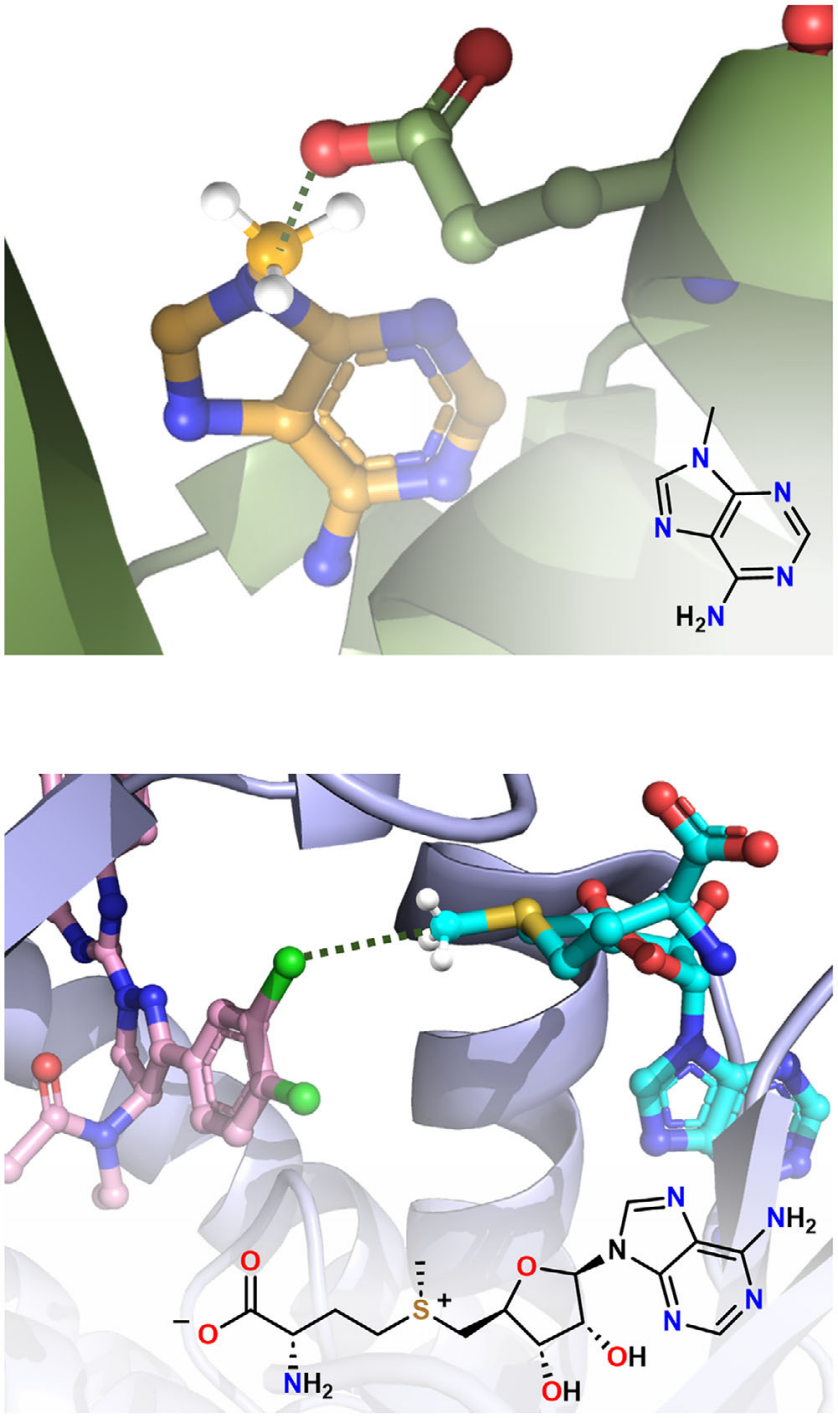
Partial view of the complex: between a ribosome‐inactivating protein and 6‐methyladenine (structural formula in the bottom‐right corner) evidencing C⋯O TtB between the CH_3_ moiety and the Glu160 C═O side chain (PDB code 1MRJ) (top); between SMYD3 inhibitor and *S*‐adenosylmethionine (structural formula at the bottom) evidencing the C⋯Cl TtB between the CH_3_ moiety and the inhibitor (PDB code 5ARG) (bottom). TtBs are black dotted lines.

**Figure 21:**
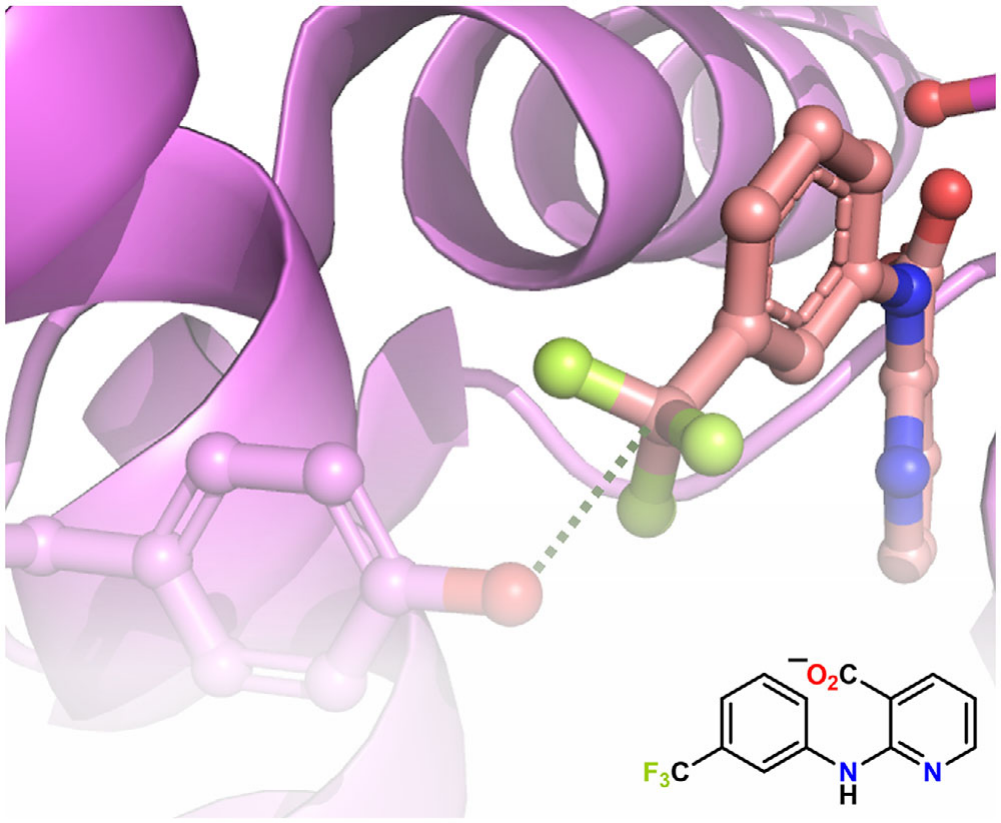
Partial view of the complex between NmrA‐like protein and 2‐{[(3‐trifluoromethyl)phenyl]amino} nicotinic acid (PDB code 2WM3) evidencing C⋯O TtB (black dotted line) between the CF_3_ group of the ligand and Tyr 246 of the protein.

σ‐Hole TtBs at C have been considered a preliminary stage of S_N_2 reactions^[^ ^225^ ^]^ and are impacting in fields as different as hydrophobic interactions^[^ ^83^ ^]^ and the bindings of ligands to nucleophilic atoms in the target protein cavity.^[^ ^228^ ^]^ For instance, the methyl groups appended to strong electron withdrawing residues in compounds which are bonded to a protein form short, linear, and attractive contacts with lone pair possessing atoms present in the binding cavity.^[^ ^85^ ^]^ This is the case, for instance, for the nucleobases,^[^ ^228^ ^]^ *S*‐adenosylmethionine,^[^ ^234^ ^]^ and acetylcholine (Figure 20).^[^ ^235^ ^]^ The propensity of organic derivatives to form σ‐hole TtBs involving carbon atoms in biological environment is confirmed by the fact that also the CF_3_ groups of several drugs form CF_3_···O TtBs when sitting in protein cavities (Figure 21). Among others, such an interaction plays an important role in the inhibition mechanism of NADP^+^‐dependent isocitrate dehydrogenase (IDH) enzyme that converts isocitrate to α‐ketoglutarate.^[^ ^229^ ^]^

The triel bond (TrB)^[^ ^59^, ^60^ ^]^ and the noble gas bond (NgB),^[^ ^96^ ^]^ namely, the interactions wherein group 13 and 18 elements act as electrophiles, are less impacting than the TtB, but few lines will be devoted also to these interactions to document the profitable use of these interactions and of the terminology that names them by referring to the group of the electrophilic atom.

The adducts formed by neutral TrY_3_ derivatives (Tr = group 13 element, Y = monovalent residue) with nucleophiles have been considered π‐hole bonded adducts and named triel bonded adducts;^[^ ^236^ ^]^ they will be discussed in Section 3.1. As examples of triel bonded systems, we discuss here the supramolecular anion···anion adducts formed by tetrahalide anions TrX_4_^−^ (X = F, Cl, Br).^[^ ^59^, ^60^ ^]^ In solid, these anions adopt a tetrahedral geometry and assemble into discrete or infinite adducts via the formation of short contacts between the halogen of a TrX_4_^−^ unit and the triel of another one. The σ‐hole nature of these contacts is supported by computational studies and confirmed by their geometry.^[^ ^237^ ^]^

**Figure 22:**
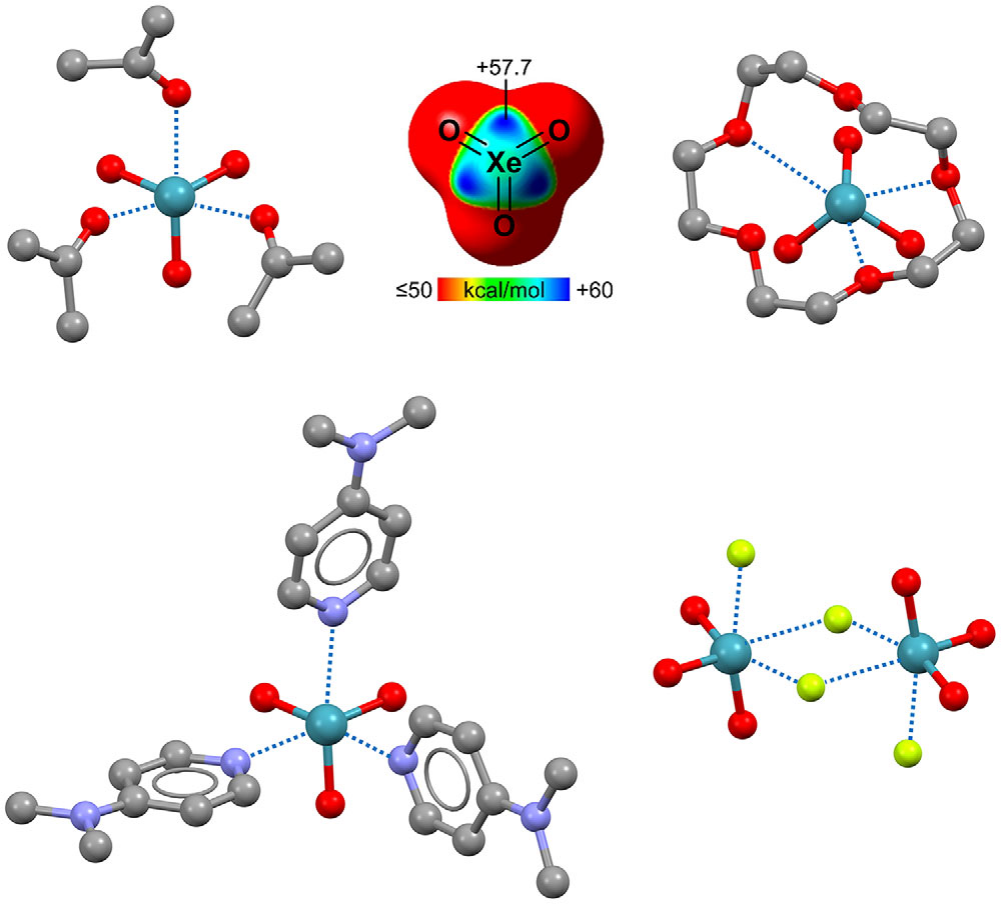
MEP surface of XeO_3_ (PBE0‐D3/def2‐TZVP level of theory) and ball‐and‐stick representation of XeO_3_ adducts with acetone (top left), 15‐crown‐5 ether (top right), 4‐dimethylaminopyridine (bottom left), and fluoride anions (bottom right).^[^ ^239^, ^240^, ^241^, ^242^ ^]^

Also, NgB can drive the self‐assembly of anion···anion adducts, for instance, in the infinite chain formed in the solid by XeF_5_^−^.^[^ ^237^, ^238^ ^]^ Importantly, XeO_3_ can act as tridentate NgB donor and form cocrystals with a variety of donors of electron density^[^ ^239^, ^240^, ^241^, ^242^, ^243^ ^]^ (Figure 22). Solid XeO_3_ detonates when thermally or mechanically shocked, whereas several of its cocrystals (e.g., XeO_3_·15‐crown‐5, (XeO_3_)_2_·(C_5_H_5_N─O)_3_, and XeO_3_·[(C_6_H_5_)_3_P═O]_2_) do not.

It is quite common that the metal centre of transition metal derivatives binds to electron density donors. The structure and stability of the resulting systems as well as the nature of the bonds driving their formation are important topics in organometallic and metalorganic chemistry. In many systems, the length and strength of these bonds are similar to the bonds in the uncomplexed parent transition metal derivatives. These bonds are typically named coordination or covalent bonds (or coordinative covalent bonds).^[^ ^244^, ^245^ ^]^

In some other systems, the bond separation is close to the sum of van der Waals radii of interacting atoms, the bond strength is smaller than that of other bonds formed by the metal, and equally important, it is possible to identify unequivocally the metal centre as the electrophilic site of the interaction. These longer and weaker bonds formed by d block elements present numerous experimental and theoretical similarities with the interactions formed by p block elements and discussed above in this section and in Sections 2.3 and 2.4. These similarities were acknowledged also by the nomenclature used to designate them as these bondings are frequently named via a specific term referring to the group of the electrophilic d block element. This was the case for the bonds formed by the elements of groups 5, (erythronium bond, EyB),^[^ ^246^ ^]^ 6 (wolfium bond, WfB),^[^ ^247^ ^]^ 7 (matere bond, MaB),^[^ ^248^ ^]^ 8 (osme bond, OmB),^[^ ^97^ ^]^ 11 (regium bond, RiB),^[^ ^249^, ^250^, ^251^, ^252^ ^]^ and 12 (spodium bond, SpB).^[^ ^253^ ^]^ These terms are convenient to differentiate noncovalent, longer, and weaker interactions from classical coordination bonds.

**Figure 23:**
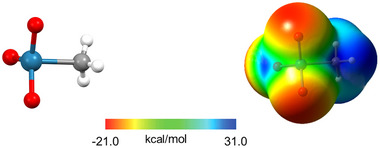
Ball‐and‐stick representation (color code: red, oxygen; imperial blue, rhenium; grey, carbon; whitish, hydrogen) and MEP surface of methyltrioxorhenium.^[^ ^254^ ^]^

**Figure 24:**
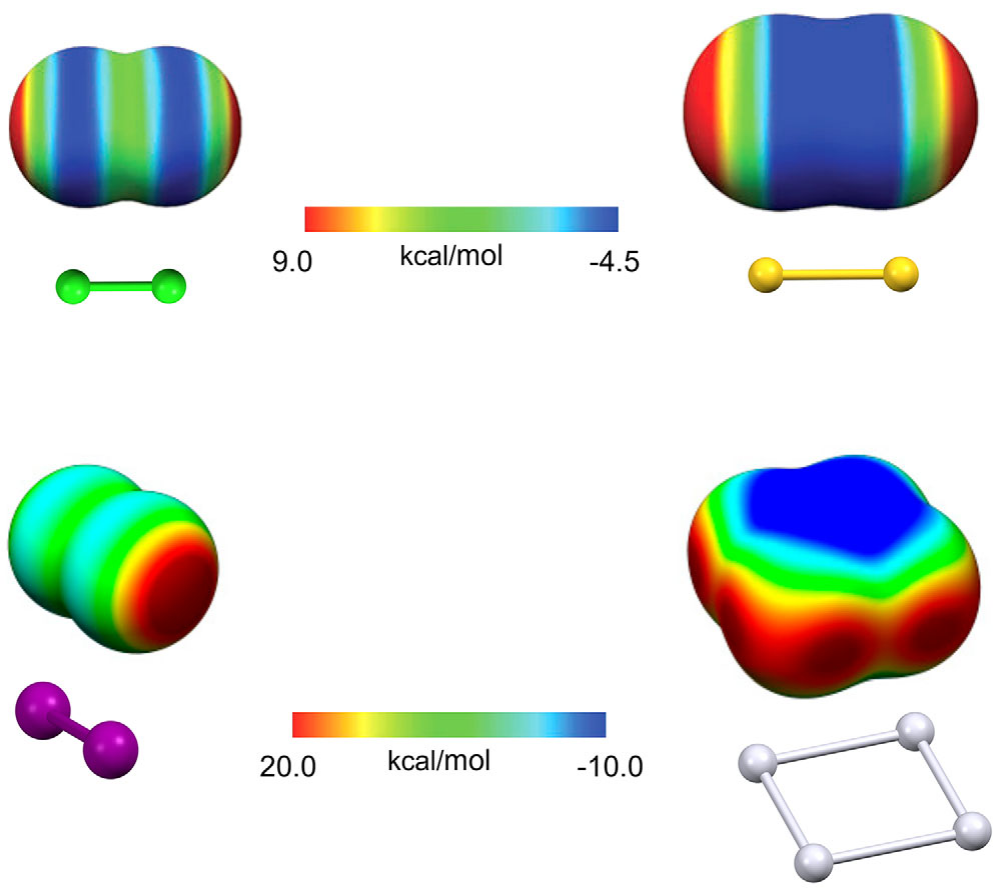
Ball‐and‐stick representations (color code: green, chlorine; yellow, gold; purple, iodine; light grey, platinum) and MEP surfaces of Cl_2_ (top left), Au_2_ (top right), I_2_ (bottom left), and Pt_4_ (bottom right).^[^ ^249^, ^255^ ^]^

The d block elements acting as electrophiles in these longer/weaker bondings show, at their surface, an anisotropic distribution of the electron density similar to that shown by the p block elements. For instance, σ‐holes are present on transition metals opposite to the σ covalent bonds between the transition metal and other elements (Figure 23)^[^ ^254^ ^]^ as well as between two metal atoms (Figure 24).^[^ ^249^, ^255^ ^]^ This latter feature is particularly important as it determines the presence of σ‐holes at the surface of metal nanoparticles. These σ‐holes are binding sites for Lewis bases, the binding energies correlates with the value of the electrostatic potential at the holes, and the catalytic properties of metal nanoparticles increase with increased affinities of reactant molecules to the holes.^[^ ^256^ ^]^

**Figure 25:**
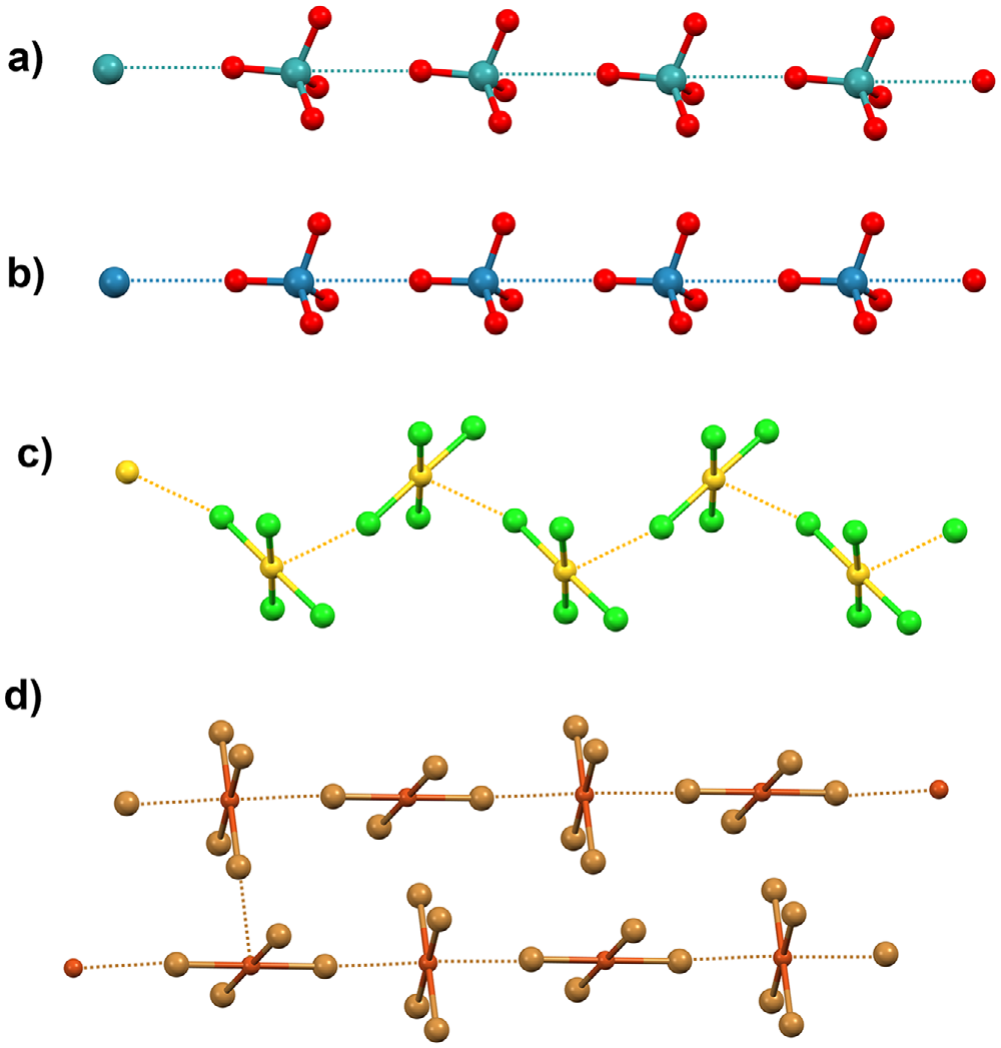
Ball‐and‐stick representation of: infinite chains formed by a) TcO_4_^−^, (Refcode VISXII), and b) ReO_4_^−^, (Refcode NAPVUZ); ribbon formed by c) AuCl_4_^−^, (Refcode UVIYOQ); (4,4) coordination network formed by d) CuBr_4_^2−^, (Refcode JEPLAR). Cations have been omitted; hole interactions are dotted lines. Color code: red, oxygen; teal, technetium; imperial blue, rhenium; green, chlorine; yellow, gold; brown, bromine; orange, copper.

The reliability of some longer/weaker bondings formed by d block elements is proven by the numerous anion···anion adducts formed by these bondings. For instance, the matere bond assembles pertechnetate (TcO_4_^−^)^[^ ^257^ ^]^ and perrhenate (ReO_4_^−^)^[^ ^248^ ^]^ anions into infinite chains and the regium bond assembles tetrachloridoaurate^[^ ^258^ ^]^ and ‐cuprate anions^[^ ^259^ ^]^ as well as their tetrabromido analogues into ribbons and (4,4) nets (Figure 25).

**Figure 26:**
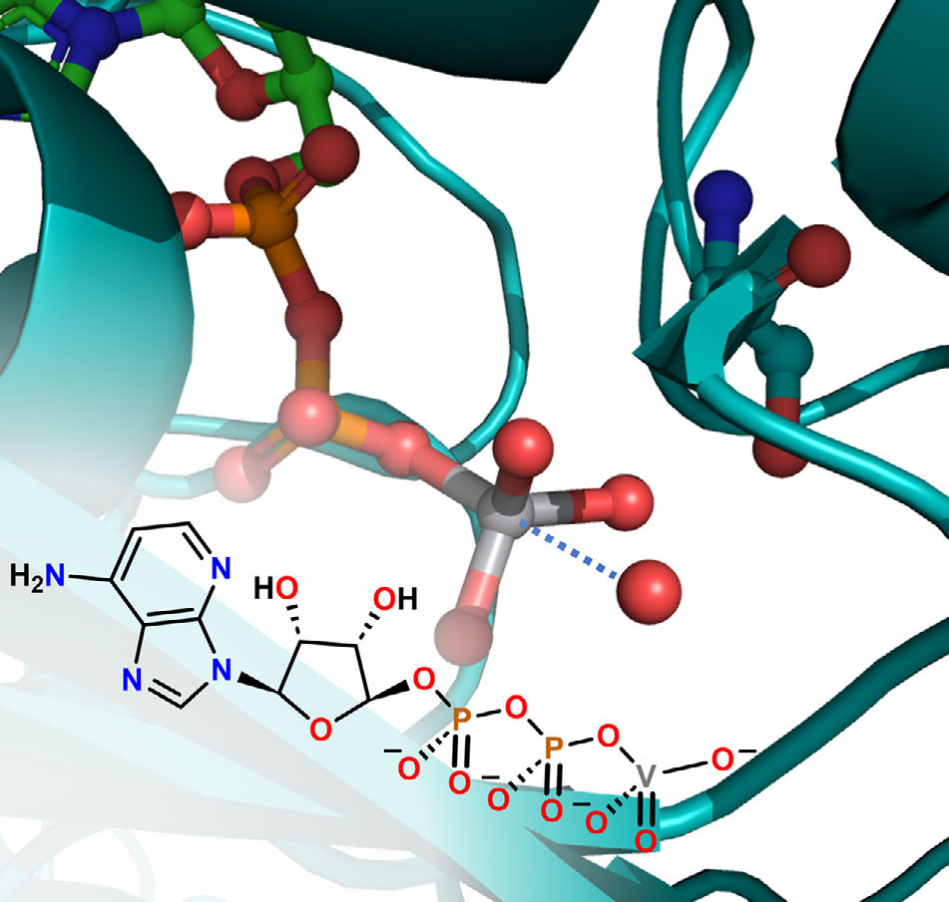
Partial view of the myosin‐II‐GGG motor domain (PDB code 7B19) evidencing the V⋯O EyB (dotted blue line) between the metavanadate moiety of ADP‐vanadate and a water molecule.

Other interesting examples of these longer/weaker bonds formed by d block elements are the interactions that drive/affect the ligand–protein binding.^[^ ^260^, ^261^ ^]^ Spodium bond has been identified in some Zn‐dependent metalloenzymes,^[^ ^262^ ^]^ and wolfium bond in the cofactor molybdopterin.^[^ ^247^ ^]^ Adenosine diphosphate vanadate (ADP‐vanadate, an adenosine triphosphate (ATP) analogue wherein a vanadate moiety substitutes for the terminal phosphoric group) has attracted considerable attention due to its ability to mimic the ATP activity and to modulate the enzymatic activity of proteins involved in cellular processes as important as signal transduction, phosphorylation, and energy metabolism. When bonded to some of these proteins, ADP‐vanadate forms erythronium bonds with oxygen atoms (Figure 26).^[^ ^263^ ^]^

## 3 Toward a Systematic Terminology of Electrophile···Nucleophile Interactions

*Plurality must never be posited without necessity* ^[^ ^264^ ^]^

William of Ockham's statement popularized by J. Punch^[^ ^265^ ^]^ as:

*Entities must not be multiplied beyond necessity*

Section 2 describes various electrophile···nucleophile interactions that have been named referring to the group of the electrophilic atom. It can be surmised that the approach is applicable also to the long/weak interactions wherein elements of groups not discussed above are the electrophile, and in this respect, it lends itself to a broad generality. In order to develop a chemical interactions taxonomy wherein the key components are the HaB, ChB, PnB, and other interactions having analogous features and whose names refer to the group of the electrophile, it may be useful: i) to identify the specific features referred to by other terms commonly used for electrophile···nucleophile interactions, ii) to relate these terms to each other and iii) to discuss their respective pros and cons.

### 3.1 Short and Reasoned Inventory of Some Terms Used for Electrophile···Nucleophile Interactions

After the mid‐20th century, when R. S. Mulliken proposed the model of the donor–acceptor complexes,^[^ ^77^, ^78^ ^]^ many systems now considered as halogen, chalcogen, or pnictogen bonded adducts were understood as complexes formed under control of charge‐transfer phenomena.^[^ ^138^, ^150^ ^]^ Now, it is commonly accepted that charge‐transfer phenomena are the origin of one of the stabilizing forces allowing for these bondings formation. Typically, the charge‐transfer importance is higher for shorter interactions and lower for longer ones; its relevance, with respect to other components, e.g., electrostatic, polarization, dispersion, and also covalent depends on the nature of the donor and the acceptor. Naming an interaction a charge‐transfer bonding is, therefore, convenient when charge‐transfer is the origin of the prevailing force that allows for its formation or when the purpose is to draw attention to this component, in other cases, the use of this term may be misleading as the attention is drawn to a minor aspect of the interactions rather than to the major one(s). The same applies to all other names that refer to a specific characteristic, be it geometric, electronic, or of any other type.

**Figure 27:**
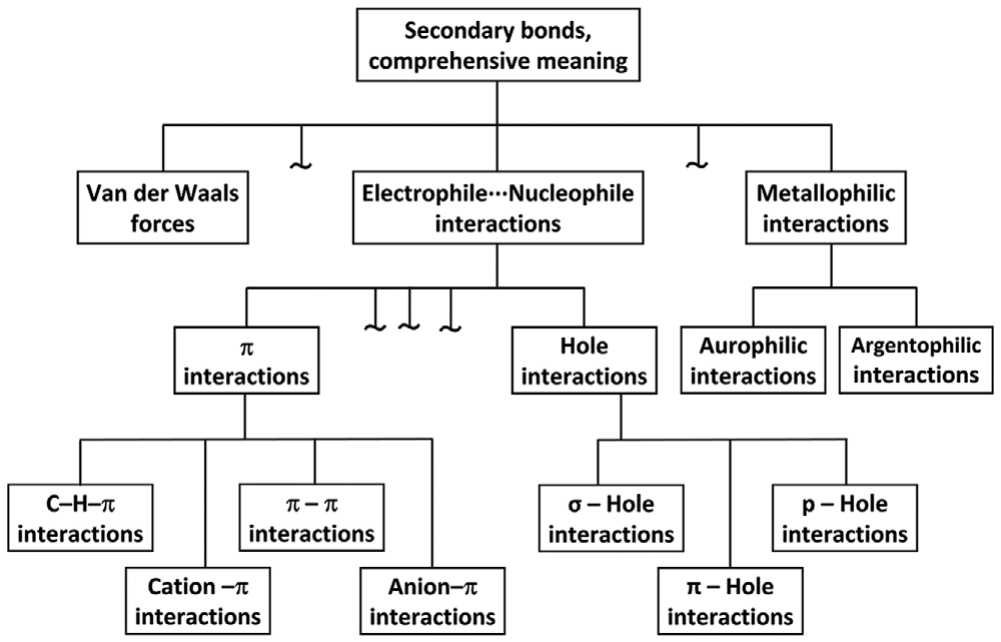
Tree notation of the hierarchical organization of some terms used to designate bondings.

The strong bonds (e.g., the covalent and coordination bonds that assemble atoms in molecules) are sometimes named primary bonds and the weak bonds (e.g., HB and HaB, which assemble molecules into nano‐ and microsized systems) are frequently named secondary bonds. This latter term “describes interactions that result in interatomic contacts that are longer than covalent single bonds, but shorter than the sum of van der Waals radii”.^[^ ^55^ ^]^ According to this comprehensive meaning, the term secondary bond clearly encompasses any type of the weak bonds independent of other features, which may differ from one type to the other (Figure 27). For instance, H.‐B. Yang indicates with this term hydrophobic and hydrophilic interactions, van der Waals adducts, HB, CH−π and π−π interactions, and even host−guest interactions.^[^ ^266^ ^]^ Variations of the term have also been used with the same meaning, e.g., secondary interactions, secondary bondings, secondary bonding interactions.

**Figure 28:**
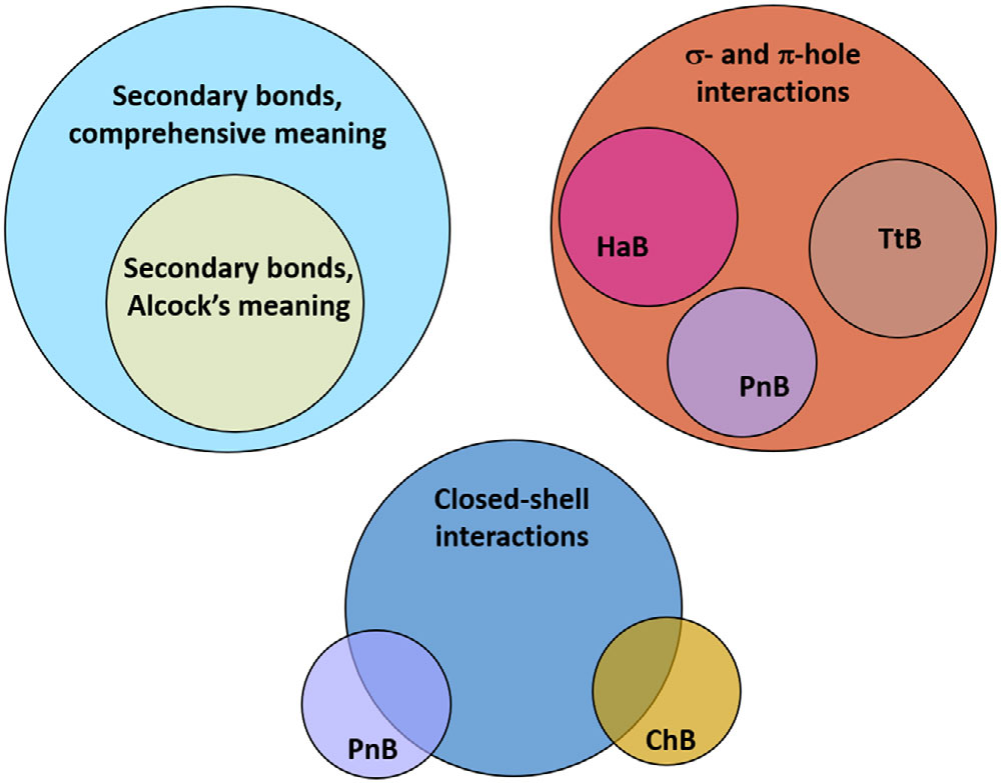
Representation according to the Venn notation showing how: Alcock's secondary bonds are a subset of the secondary bonds according to the comprehensive meaning (top, left); HaB, ChB, and PnB are subsets of the σ‐ and π‐hole bonds set (top, right); the ChB and PnB intersect with the closed‐shell interactions (bottom) (e.g., SF_4_, Martin reagent, and SbCl_5_^2−^ have more than eight valence electrons and form ChBs and PnBs, respectively).

N. W. Alcock proposed in 1972 to use the term secondary bonding for a much more limited set of interactions sharing some geometric features in the solid phase.^[^ ^54^ ^]^ Specifically, Alcock proposed that the secondary bonding in X─E···N is the weak E···N bond that develops approximately on the extension of the strong X─E bond (E = groups 14–18 element bonded to X via a strong (primary) bond; N = electron rich atom, typically a lone pair possessing atom, X─E···N angle ≈ 180°). Alcock's secondary bondings are a subset of the secondary bonds according to the comprehensive meaning considered above (Figure 28, top left) and encompass the tetrel, pnictogen, chalcogen, halogen, and noble gas bonds, which develop opposite to a σ covalent bond, but not those that develop orthogonal to the group 14–18 elements nor the analogous interactions formed by the group 13 elements (triel bonds) or by the elements of the d‐block (e.g., matere^[^ ^58^ ^]^ and osme^[^ ^97^ ^]^ bonds). In other words, the set of Alcock's secondary bonds intersects with the sets of the tetrel, pnictogen, chalcogen, halogen, and noble gas bonds and is disjoint from the sets of the triel bond and analogous interaction wherein the electrophile is a d‐block element (Figure 1, bottom).

The wording secondary bond and its variations have been and are used with both the more comprehensive meaning discussed first and the more limited one discussed second. The interaction features implied and communicated after the two meanings are quite different. The use of this wording thus lends itself to a certain ambiguity if a reference, or a description, is not added in order to specify in what sense the wording is used.

Most HaBs and other analogous interactions are closed‐shell interactions.^[^ ^71^, ^72^, ^73^, ^267^, ^268^ ^]^ For instance, when the halogen of ArX, the chalcogen of Ar_2_Ch, or the pnictogen of Ar_3_Pn form the adduct Ar_n_–E···N (E = halogen, chalcogen, pnictogen; *n* = 1, 2, 3; N = nucleophile), the atom E has already satisfied its valency in the Ar*_n_*–E molecular entity, and according to the simple arguments of Lewis bonding theory, may not be expected to need more bonding electrons, i.e., to form additional bonds. Indeed, closed‐shell interactions are widely documented in the structural chemistry of heavy main‐group elements since long ago.^[^ ^76^, ^269^ ^]^ The understanding of HaB, ChB, PnB, etc. and their bonding modes via the tools developed within the frame of the closed‐shell interactions has not been fully explored to now, but it may offer new useful insights thanks to the wide commonalities between the sets of bondings.

The tendency of the closed‐shell atom to accept electron density from a nucleophile can be moderate and the separation of formed bonding is close to the sum of van der Waals radii of involved atoms. These bondings are the typical HaBs, ChBs, PnBs, etc. But for some atoms, frequently the heavy main‐group elements, the tendency to accept electron density can be remarkably strong, to the point that the bond length is close to a covalent bond formed by involved atoms and an “expansion of the valence shell” occurs. The concept of hypervalency has long been debated and a rigorous definition can be hardly given, but in our context it can be considered that the atom E forms a hypervalent bond when, doing so, it gets surrounded by more than four pairs of valence electrons.^[^ ^31^ ^]^ Similarities between the closed‐shell and hypervalent bonds may be envisaged.^[^ ^31^ ^]^ The moderate donation of electron density from the electron rich site to the electrophilic closed‐shell atom (e.g., the n→σ* donation after Mulliken's notation) that occurs in most TtBs, PnBs, ChBs, and HaBs may be viewed as the initial stage of the expansion of the octet. Indeed, known adducts show that there is a continuum of bond lengths and strengths between the prototype closed‐shell bonds and the prototype hypervalent bonds, namely, between the longest/weakest and the shortest/strongest bonds. For instance, the nearly symmetric I_3_^−^ anions, wherein the two I─I bonds separations are close to the covalent bond in I_2_, can be considered as prototype cases of hypervalent bonds and are typically modeled as 3‐centre‐4‐electron systems. Differently, the asymmetric I_3_^−^ anions, wherein an I_2_ unit interacts with an I^−^ anion via an I···I contact that is marginally shorter than two times the iodine van der Waals radius and which develops on the extension of the I_2_ covalent bond, can be considered as prototype cases of closed‐shell interactions, specifically of HaBs. There are reasons for rationalizing the intermediate systems (i.e., the I_3_^−^ anions with intermediate separation values) via both models, and for designating them via both terminologies as well as halogen bonded adducts. Similar relationships exist among other interactions named by referring to the group of the electrophile (e.g., PnB) and the closed‐shell interactions as well as the hypervalent bonds.

The σ‐^[^ ^43^, ^44^, ^45^, ^46^, ^47^, ^48^ ^]^ or the π‐hole^[^ ^49^, ^50^, ^51^ ^]^ bonds are the attractive interactions that nucleophiles form with σ‐ or π‐holes, i.e., the regions with depleted electron density, and typically with positive electrostatic potential that bonded atoms have on their surface opposite to σ covalent bonds or above and below an atom adopting a planar geometry and forming a π bond. The HaB and other analogous electrophile···nucleophile interactions described in Section 2 are subsets of the σ‐ and π‐hole bonds set (Figure 28, top right).^[^ ^270^, ^271^ ^]^

The σ‐hole presence opposite to a σ covalent bond can be explained by considering that when a mono‐occupied p (or sp, sp^2^, or sp^3^) orbital of an atom forms a σ covalent bond, the electron localizes preferentially in the orbital lobe between the two covalently bonded atoms. A deficiency of electron density occurs in the other lobe (opposite to the covalent bond) and the σ‐hole is the region occupied by this lobe at the outer surface of the atom. Similarly, a π‐hole develops on an atom due to the relocalization of the electron of a mono‐occupied atomic orbital that forms a molecular π covalent bond with a more electron withdrawing atom. The presence of σ/π‐holes on bonded atoms surface is a general phenomenon and the images of MEP surfaces effectively visualize the regions with electrophilic or nucleophilic character and their depth. This favoured the rapid success of the σ/π‐hole bonds model and terminology.

The σ/π‐hole model identifies interactions by localizing the σ/π‐holes in the starting component and by considering the resulting electrostatic component of the attractive forces responsible for the interaction formation. As it is the case for other terminologies that refer to a single bonding component, some problems may arise in the use of the terms σ‐hole bond and π‐hole bond. For instance, it may happen that σ/π‐holes are not found on bonded atoms which nevertheless form, with nucleophiles, short attractive interactions whose features are those of σ/π‐hole bonds. This typically occurs when the interacting electrophilic and nucleophilic atoms are in the same molecule and their forming an intramolecular short contact “buries” the hole inside the molecule.^[^ ^272^ ^]^ It is obviously hard to name these short contacts σ/π‐hole bonds (these terms asking by definition for the presence of σ/π‐holes); differently, if they are named by referring to the electrophilic atom (e.g., HaB, ChB, etc.) their analyzes based on convenient theoretical models consistently identify their bonding features. For instance, the charge‐transfer model may identify the typical overlap between an occupied orbital of the nucleophile and an empty orbital on the electrophile and the transferred electron fraction can be calculated. An analysis of the electron density may pinpoint a bond path connecting the interacting atoms and identify other fingerprints of attractive interactions.

The σ/π‐hole bond model analyzes the MEP of the starting modules. In some cases, molecular entities that are not electrophilic as isolate species, attractively interact with nucleophiles in solution or in the solid as a consequence of the polarization induced by nearby molecular entities. In other words, the electrophilic character of some molecular species is switched on by the redistribution of its electron density induced by surrounding entities, not by the electron relocalization associated with σ or π bond formation. This is typically the case when a variety of oxyanions self‐assemble into anion···anion adducts under control of the HaB or other analogous interactions (e.g., ChB^[^ ^273^ ^]^ and MaB). For instance, IO_4_^−[^ ^155^ ^]^ and ReO_4_^−[^ ^248^ ^]^ anions have a negative electrostatic potential on their entire surface and the same happens for the IO_3_^−[^ ^154^ ^]^ anion. No σ‐holes are detected on the surface of the isolate IO_3_^−^ anions and the MEP maximum is located at the lone‐pair, but the MEP of ammonium iodate (a model for the ion pairs present in iodate salts) shows three well defined σ‐holes with positive potential on I at the extension of the O─I covalent bonds. In crystalline iodate salts, these positions form short O─I···O HaBs with the negative and nucleophilic O atoms of nearby anions and 1D or 2D supramolecular anionic networks are formed.

**Figure 29:**
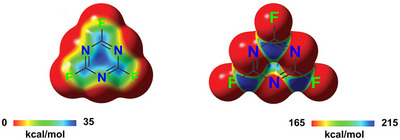
MEP surface of trifluorotriazine at: the standard 0.001 a.u. isodensity value evidencing one hole in the ring center and three holes over the carbons (left); a much higher isodensity (0.05 a.u.) evidencing the convergence point of three σ‐holes of the C─F bonds.

More important, some interactions have been considered as π‐hole bonds by some authors and as σ‐hole bonds by some others. For instance, the regions above and below the centre of hexafluorobenzene ring have positive electrostatic potential and attractively interact with nucleophiles. It is well‐known that anions approach these regions from directions orthogonal to the ring plane; these bonds are typically named anion···π interactions,^[^ ^274^ ^]^ and, considering their geometry, they have also been called π‐hole bonds.^[^ ^152^ ^]^ But in C_6_F_6_, the six C─F covalent bonds form six σ‐holes directed toward the ring centre. In this position, the six σ‐hole coalesce into a single hole, which, although being in the ring plane, radiates beyond the π‐electrons cloud and determines the positive electrostatic potential at the molecular surface.^[^ ^275^ ^]^ It is here important to note that genuine π‐holes are not in the ring plane (as in C_6_F_6_), but above and below, the ring plane. For instance, this is the case in hexaoxocyclohexane (C_6_O_6_) whose hole above and below the centre ring originates from the coalescence of the six π‐holes of the carbonyls. In C_6_F_6_, the molecular geometry prevents nucleophiles from approaching any single σ‐hole from a direction opposite to the respective C─F bond, the only possible approach direction of nucleophiles being orthogonal to the ring plane. Considering their geometry, the anion···π interactions formed by C_6_F_6_ should be named π‐hole bonds. But this term is misleading in relation to the nature of the involved holes as, on considering this feature, these interactions should be named pseudo‐π‐hole bonds or, more descriptively, orthogonal‐σ‐hole bonds. Other aromatic molecules behave similarly,^[^ ^276^ ^]^ for instance, trifluorotriazine, which, when the distribution of the electron density is analyzed at the convenient isosurface, reveals the presence of a σ‐hole at the ring centre and in the ring plane (formed on coalescence of the three σ‐holes associated with the C─F covalent bonds) and three π‐holes above and below the carbons associated with the C═N moieties (Figure 29).

**Figure 30:**
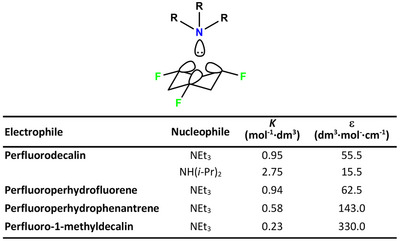
Representation of the perfluorocyclohexane/amine complex evidencing the overlap of amine lone pair and the coalescence region of one lobe of the σ* bonds of the equatorial C─F bonds (only three fluorine atoms of perfluorocyclohexane are reported). The table reports the equilibrium constants (*K*) and extinction coefficients (*ε*) of some charge‐transfer complexes.^[^ ^277^ ^]^

The coalescence of σ‐holes at the ring centre can drive the formation of orthogonal σ‐hole bonds also in aliphatic systems. Cyclic perfluoroalkanes form charge‐transfer complexes with unhindered amines as revealed by the appearance of new UV bands at 260–270 nm.^[^ ^277^ ^]^ This behaviour has been explained by considering that one lobe of the σ* orbitals of equatorial C─F bonds in position 1, 3, 5 of perfluorocyclohexanes is oriented toward the ring centre and overlaps and coalesces in the regions above and below the ring (Figure 30). These lobes of σ* orbitals localize in the same regions of the carbon σ‐holes generated by corresponding C─F bonds and their coalescence corresponds to the coalescence of the three σ‐holes on carbon atoms in positions 1, 3, 5. The tendency to accept electron density in the region of orbitals coalescence is enhanced and drives the approach of the amine lone pair after a direction orthogonal to the ring plane.

Misleading messages deriving from conflicting geometric and orbital features are given also when the term π‐hole bond is used for some interactions formed by group 13 derivatives. The bonds that neutral TrY_3_ derivatives (Tr = group 13 element, Y = monovalent residue) form with nucleophiles are orthogonal to the molecular plane and have therefore been named π‐hole bonds.^[^ ^236^ ^]^ But the electrophilicity of the group 13 element does not originate from a π‐hole associated with the formation of a π covalent bond, but from an empty p orbital. The charge transfer from the nucleophile is not of n→π* type (as when nucleophiles interact with π‐hole donors and forms a genuine π‐hole bond),^[^ ^50^ ^]^ but of n→p type.^[^ ^270^ ^]^ Acknowledging the orbital characteristics, some authors have named these bonds p‐hole bonds^[^ ^275^ ^]^ and proposed the term hole bonds to encompass the terms σ‐, π‐, and p‐hole bonds (Figure 27).

M. Taylor pondered that the naming of electrophile···nucleophile interactions by referring to the group to which the electrophile belongs generates an excessively diversified nomenclature by proposing “more terms than necessary”.^[^ ^270^ ^]^ He suggested that “the simplest alternative nomenclature” is to use the terms “σ‐hole interaction, π‐hole interaction, and p‐hole interaction, as well as perhaps perihypervalent bond and pericovalent bond”.

**Figure 31:**
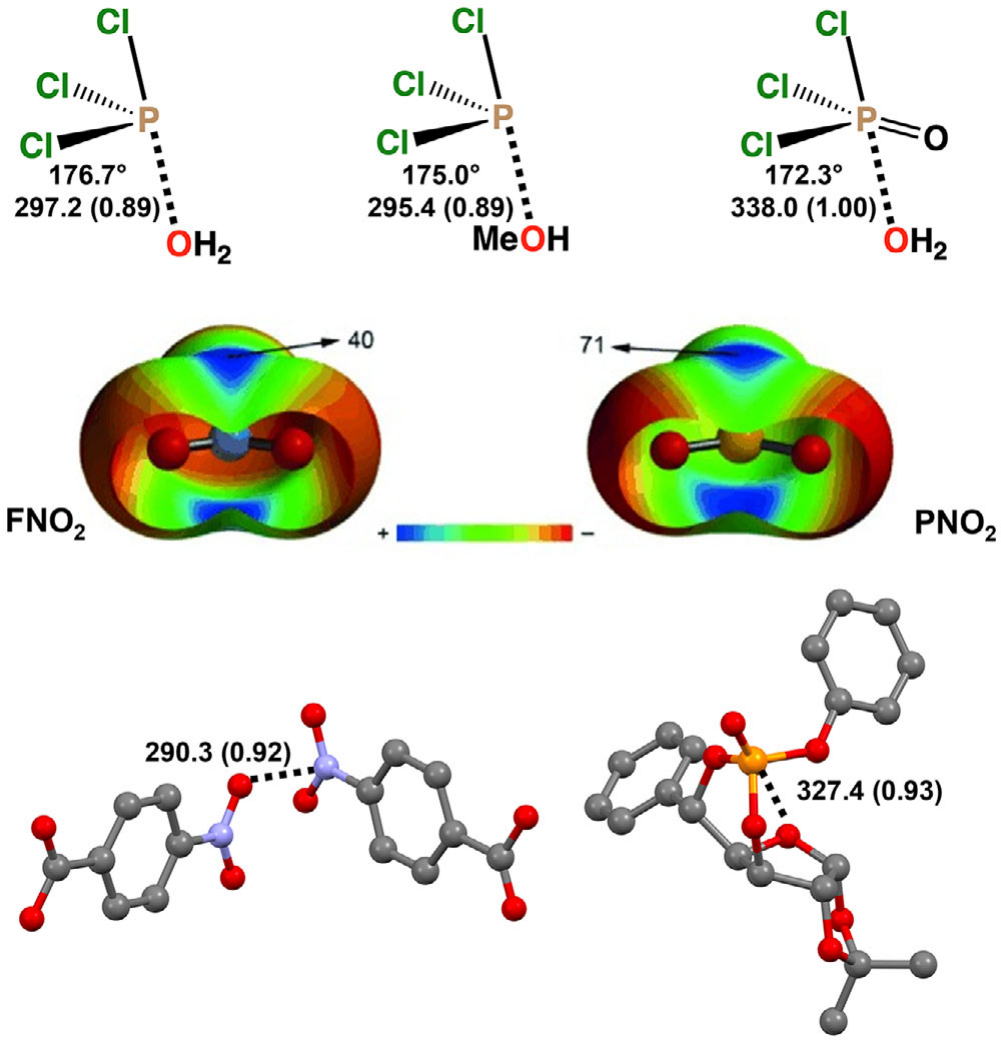
Top: Pnictogen bonded adducts identified via their IR spectra;^[^ ^278^, ^279^, ^280^ ^]^ computed values of P···O bondings lengths (pm, Nc values in parenthesis) and Cl–P···O angles (°) are reported. Mid: MEPs of FNO_2_ and FPO_2_ with energies at the π‐holes (kcal mol^−1^, B3LYP/6‐31+G* level of theory;^[^ ^51^ ^]^ Bottom: ball‐and‐stick representation of 4‐nitrobenzoic acid^[^ ^281^ ^]^ (Refcode AJAKEB) and of xylofuranose phosphate derivative^[^ ^282^ ^]^ (Refcode AHUDAI) evidencing the N/P···O PnBs and respective lengths.

**Figure 32:**
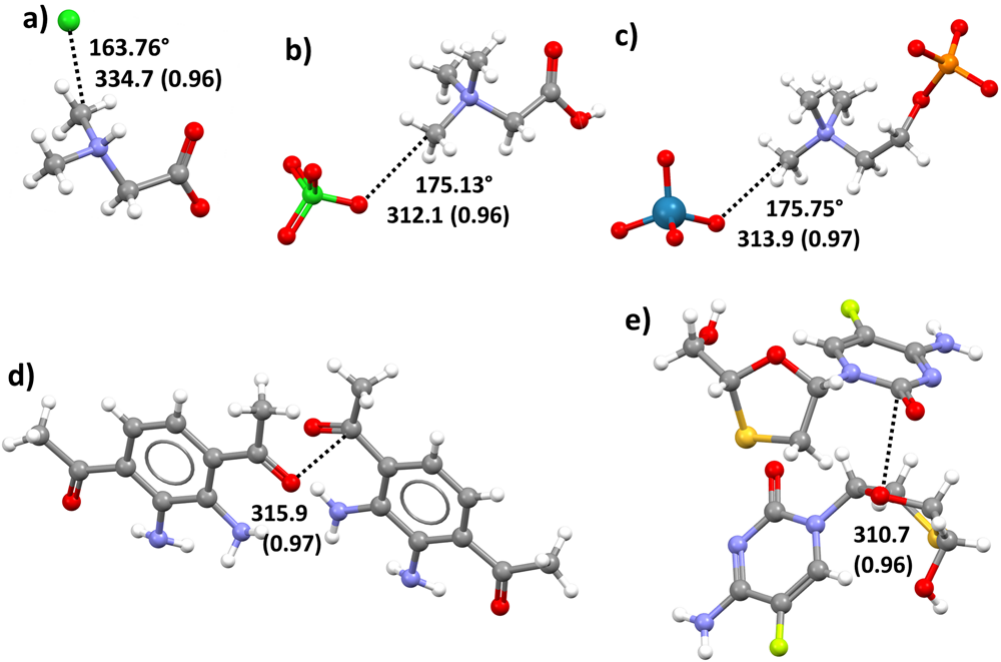
Ball‐and‐stick representation of: a), dimethylglycine chloride^[^ ^284^ ^]^ (Refcode CIVKAW01); b), betaine perchlorate^[^ ^285^ ^]^ (Refcode XANDOF); c), phosphocholine^[^ ^235^ ^]^ perrhenate (CCDC number 2259081); d), 1,2‐diamino‐3,6‐diacetylbenzene, a green light emitter^[^ ^286^ ^]^ (Refcode RODCOG); e), emtricitabine, an antiviral drug for human use^[^ ^287^ ^]^ (Refcode TEVYIF). σ‐Hole TtBs in (a) – (c) and π‐hole TtBs in (d) and (e) are dotted black lines. Bondings lengths (pm, Nc values in parenthesis) and angles (°) are reported. Color code: grey, carbon; whitish, hydrogen; red, oxygen; blue, nitrogen; green, chlorine; imperial blue, rhenium; orange, phosphorous; yellowish green, fluorine; ocher, sulfur.

The terminology that names electrophile···nucleophile interactions by referring to the group of the electrophile proposes umbrella terms that indicate with a single name the interactions formed by all the elements of a group and adopt the geometry of both σ‐ and π‐hole bonds. Examples of this characteristic are given in Figure 5 for HaBs formed by bromine and in Figure 31 for PnBs formed by nitrogen and phosphorous. Figure 32 gives analogous cases for TtBs formed by carbon and shows that also the interactions wherein nucleophiles approach a carbonyl moiety adopting a Bürghi–Dunitz angle are TtBs.^[^ ^51^, ^218^, ^219^ ^]^

The dative bond is defined as the “coordination bond formed upon interaction between molecular species, one of which serves as a donor and the other as an acceptor of the electron pair to be shared in the complex formed”.^[^ ^283^ ^]^ Clearly dative bonds are electrophile···nucleophile interactions and the example given in the IUPAC definition is the H_3_B···NH_3_ adduct, namely, a system that, according to its geometry, might be considered a p‐hole bonded adduct. However, the systematic extension of the “hole bond” concept and naming, from cases wherein the hole presence is the result of the electron relocalization occurring when a σ or π covalent bond is formed (as it is for genuine σ‐ and π‐hole bonds) to systems wherein the hole is an empty atomic p orbital (as it is in the H_3_B···NH_3_ adduct) would lead to hardly acceptable results. For instance, the chlorous acid should be represented as HOCl···O.^[^ ^275^ ^]^

Some features of the interactions of different sets named by referring to the group of the electrophile, typically the most basic features, are the same for all sets, e.g., the presence of a region of depleted electron density on the electrophilic atom. When naming these interactions, if the aim is to stress these basic common features, a term more general than that referring to the group of the electrophile can be used, e.g., σ‐hole bond instead of HaB or TtB (Figures 27 and 28). Some other interactions features vary from one set, namely, group, to the other. For instance, the deviations from linearity of σ‐hole HaBs is typically smaller than that of σ‐hole ChBs which, in its turn, is smaller than that of σ‐hole PnBs.^[^ ^43^ ^]^ Another example is the propensity of the different elements of a given group to act as the electrophile in a σ‐hole bond. This propensity increases with the molecular weight when the electrophilic element is from groups 15, 16, and 17. Group 7 elements behave similarly, whereas group 5 elements show an opposite trend. In the CSD, the fraction of structures containing a permanganate, pertechnetate, and perrhenate unit and forming a matere bond is smallest for permanganate, intermediate for pertechnetate, and greatest for perrhenate.^[^ ^248^ ^]^ Differently, metaniobate units, and even more metatantalate ones, are less likely to form erythronium bonded adducts than metavanadate units.^[^ ^246^ ^]^ A specific name designating the interactions wherein the elements of a given group are the electrophiles acknowledges and communicates also these specific characteristics in contrast to a more general and encompassing name that neglects these specific characteristics and acknowledges and communicates the more basic commonalities. This makes positive reasons for using specific or general names depending on the context and the target of the communication and for developing a taxonomy of chemical interactions wherein terms with different levels of generality and descriptiveness are ranked and their use is categorized.

### 3.2 Taxonomy of Electrophile···Nucleophile Interactions and Its Epistemological Aspects

As recently acknowledged, among others,^[^ ^68^, ^69^, ^70^ ^]^ in a paper resulting from a *Faraday Discussion*,^[^ ^69^ ^]^ an agreement is emerging in the scientific community for the usefulness of a hierarchical categorization of interactions. Figures 1 and 28 give such a categorization of some interactions by resorting to the Venn notation and Figure 27 by resorting to the tree notation. Some of the terms in these figures are highly general and scarcely descriptive, namely, they encompass large sets comprising many different interactions sharing only very fundamental similarities. Secondary interactions according to the comprehensive meaning may be considered as the most general term (Figure 27). Other terms are scarcely general and highly descriptive, namely, they designate quite small sets of interactions sharing similarities also in very specific features (e.g., C─H···π interactions). Some other terms have an intermediate level of generality and descriptiveness. Interactions designated by less general terms are subsets of the interactions designated by more general ones (Figures 1 and 28).

It is not uncommon that a given interaction can be described by using different names referring to a single geometric, chemical, physical, and quantum‐mechanical aspect. These terms are all useful. By referring to different aspects of the interactions, they complement each other in describing the multisided nature of interactions, but in doing so these names emphasize one feature over the others. To give well‐established examples of names referring to a specific aspect, and thus emphasizing it, we may mention the lock‐and‐key term, which refer to geometric features, or the terms 3c‐2e and 3c‐4e interactions, which refer to electronic features; other cases (closed‐shell, charge‐transfer, etc.) have been discussed above.

Such emphasis on one aspect may be partially determined by subjective opinions^[^ ^152^ ^]^ and it may communicate confusing messages as it is the case of the so named anion–π interactions.^[^ ^288^ ^]^ As discussed in Section 1, from a logic and semantic point of view, names of interactions should refer to the most important feature(s) of interactions, but the relative importance of different features may vary from one system to the other also in sets of quite similar interactions and may even depend on the approach used to study a given interaction.

A taxonomy of chemical bonds based on the recent IUPAC definitions of HB, HaB, ChB, and PnB might avoid some of the pitfalls discussed above as such a systematic nomenclature refers to one of the atoms that forms it, namely, to a feature that does not change in all the interactions of the set. In the list of bondings mentioned above, the pairing of the HB to the HaB, ChB and PnB is not only justified by the common reference of the respective names to the electrophilic site of the interaction, but, equally important, by the fact that also the HB has been rationalized via the σ‐hole bond model^[^ ^289^ ^]^ and via the charge‐transfer model.^[^ ^290^, ^291^ ^]^

The well‐established usefulness of taxonomical classification in biology^[^ ^86^, ^87^, ^88^ ^]^ promoted the use of analogous categorization approaches in quite different fields, spanning business and economics,^[^ ^89^, ^90^ ^]^ geology,^[^ ^91^ ^]^ and education.^[^ ^92^ ^]^ A taxonomy of chemical bondings will offer a set of specific terms designating interactions according to a hierarchical categorization, i.e., as a function of their organization into sets and subsets identified via commonalities with different levels of generality. In this way the level of the communicated information becomes well‐defined and the storage and management of information, notably via computer based processes, becomes easier and more effective. Moreover, a taxonomy may promote a more rigorous use of the terms and may help in polishing their meaning by favouring a clearance of ambiguities and/or misuses. Ambiguities related to the use of the term “secondary bond” and the misuse of the term “π‐hole bond” have been discussed in the details in Section 3.1; other pitfall related to the use of some terms will not be discussed as it is out the scope of this paper.

**Figure 33:**
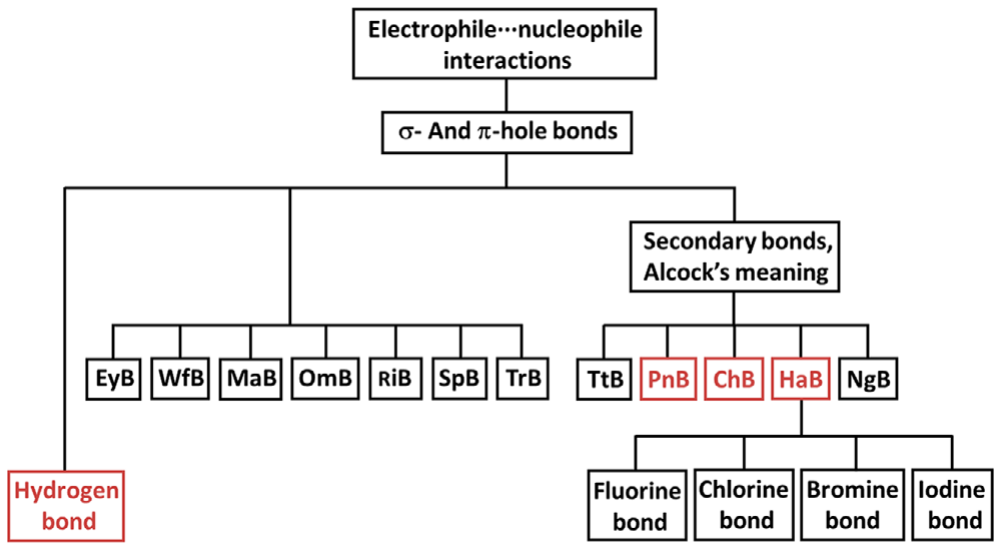
Tree notation of a chemical interactions taxonomy based on a systematic reference to the electrophile. In red, the IUPAC defined terms. Other terms could be added at different levels of generality (e.g., carbon bond^[^ ^83^, ^84^ ^]^ and selenium bond^[^ ^82^ ^]^ at the lowest level of generality where the fluorine bond and other subsets of the HaB are reported).

Figure 33 proposes a taxonomy of some electrophile···nucleophile interactions by using the tree notation. This tree comprises a term (i.e., σ‐ and π‐hole interactions) for a set characterized by a higher degree of comprehensiveness, namely a term designating a large number of bondings sharing only few and quite basic commonalities. The tree comprises also terms for sets characterized by a mid degree of comprehensiveness, namely, terms designating bonds sharing more specific features. These terms are the IUPAC defined HaB, ChB, PnB, and other ones designating the weak bonds formed by the elements of a given group and acting as electrophiles. But according to R. Descates “Comprendre c'est distinguer”^[^ ^11^ ^]^ and terms for sets characterized by a lower degree of comprehensiveness are also reported, namely, the terms referring to the element acting as the electrophile in the weak bond. The terms hydrogen bond and fluorine,^[^ ^292^ ^]^ chlorine,^[^ ^79^ ^]^ bromine,^[^ ^117^ ^]^ and iodine^[^ ^293^ ^]^ bonds are indicated in the tree thanks to their particularly common use. Other terms referring to the name of the electrophilic element are frequently used in the literature, e.g., carbon bond^[^ ^83^, ^84^ ^]^ and sulfur or selenium bonds,^[^ ^82^ ^]^ which are subsets of the TtB and ChB, respectively. These terms designate sets of interactions at the lower degree of comprehensiveness, but they have not been reported in the tree for sake of simplicity. Ockham's razor^[^ ^264^ ^]^ reminds that the multiplication of the terms is justified, limited to its usefulness. The present state of the art suggests that the nomenclature based on the name of the electrophilic atom is the lowest level of generality useful and convenient to now.

Previous sections in this paper prove that it is reasonably safe to state that a taxonomy of electrophile···nucleophile interactions, wherein the core terms refer to the group of the electrophile, is: i) descriptive (the interaction is unequivocally identified by referring to one of the atoms forming the bond), ii) comprehensive (all the interactions wherein the elements of the respective group act as electrophile are encompassed), iii) systematic (the reference is to the archetype of systematicity in chemistry, the periodic table of the elements), iv) consistent (the bond name is referring to the electrophile, similar to the hydrogen bond, the most important interaction), and iv) invariant (the understanding of interactions may evolve, the relative relevance of features may change, the elements forming them do not).

## 4 Summary and Outlook

*in the symbols which we use something is arbitrary*,

*something not (6.124)*

L. Wittgenstein^[^ ^38^ ^]^

The advancement of science is based on the accumulation of new findings. Charles Darwin's work demonstrates emblematically that proposing a hierarchical organization of physical entities (living organisms in his theory) may be as important as reporting new phenomena, both actions being crucial for formulating more likely and accurate predictions. This fact may underpin the usefulness of this paper that proposes a hierarchical organization of some terms already used to designate chemical interactions and puts forward a taxonomy that aims at being descriptive, systematic, consistent, and invariant. The targets of this organization are: i) to favour a discussion on the logic underlying the terms used to designate chemical interactions; ii) to distinguish between interactions sets characterized by different degrees of similarity; iii) to pursue an agreement on a terminology that acknowledges and declares the similarities between different interactions and the differences between similar interactions. The final aim is to be proactive in developing a common language and a “common understanding of commonly used language”^[^ ^29^ ^]^ so that new findings are communicated and stored more effectively via both human and machine based protocols.

Any bonding is the result of the balanced combination of many different measurable and computable features. Naming interactions via terms referring to one single feature may not be particularly effective for the construction of robust chemical databases. The same interaction can be given different names and to recognize the similarities and differences between differently named interactions requires sophisticated, and possibly subjective, evaluations. Automated data storage and retrieval are becoming more and more common and the taxonomy presented in this paper is highly relevant to these emerging fields. Consistent terminology and data labelling are critical for the emerging fields of machine learning in chemistry, automated knowledge extraction, and reliable algorithm training. The proposed systematic terminology that refers to invariant and nonsubjective features will benefit both human discourse and machine‐readable chemical databases.

If “in the symbols which we use something is arbitrary, something not”,^[^ ^38^ ^]^ the proposed organization and taxonomy aims at minimizing the arbitrary component in the terminology used for chemical bondings and in maximizing the nonarbitrary component. Basic and applied research in all fields related to the weak bonds may thus witness more fruitful communications and faster advancements. Vague terminology, and loose definitions, are a recipe for confusion and misunderstanding and unfortunately, this is what sometimes happened. This paper pursues some ways to jump over such hardles.

## Conflict of Interests

The authors declare no conflict of interest.

## Data Availability

The data that support the findings of this study are available from the corresponding author upon reasonable request.
